# All-Atom Photoinduced Charge Transfer Dynamics in Condensed Phase via Multistate Nonlinear-Response Instantaneous Marcus Theory

**DOI:** 10.1021/acs.jctc.4c00010

**Published:** 2024-04-24

**Authors:** Zengkui Liu, Zailing Song, Xiang Sun

**Affiliations:** † Division of Arts and Sciences, NYU Shanghai, 567 West Yangsi Road, Shanghai 200124, China; ‡ NYU-ECNU Center for Computational Chemistry at NYU Shanghai, 3663 Zhongshan Road North, Shanghai 200062, China; ¶ Department of Chemistry, New York University, New York, New York 10003, United States

## Abstract

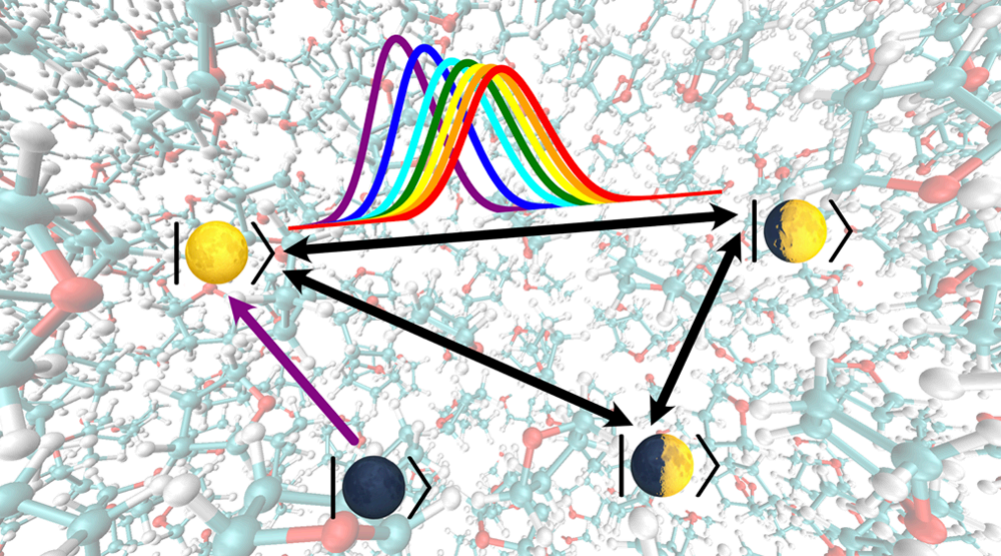

Photoinduced charge transfer (CT) in the condensed phase is an essential component in solar energy conversion, but it is challenging to simulate such a process on the all-atom level. The traditional Marcus theory has been utilized for obtaining CT rate constants between pairs of electronic states but cannot account for the nonequilibrium effects due to the initial nuclear preparation. The recently proposed instantaneous Marcus theory (IMT) and its nonlinear-response formulation allow for incorporating the nonequilibrium nuclear relaxation to electronic transition between two states after the photoexcitation from the equilibrium ground state and provide the time-dependent rate coefficient. In this work, we extend the nonlinear-response IMT method for treating photoinduced CT among general multiple electronic states and demonstrate it in the organic photovoltaic carotenoid–porphyrin–fullerene triad dissolved in explicit tetrahydrofuran solvent. All-atom molecular dynamics simulations were employed to obtain the time correlation functions of energy gaps, which were used to generate the IMT-required time-dependent averages and variances of the relevant energy gaps. Our calculations show that the multistate IMT could capture the significant nonequilibrium effects due to the initial nuclear state preparation, and this is corroborated by the substantial differences between the population dynamics predicted by the multistate IMT and the Marcus theory, where the Marcus theory underestimates the population transfer. The population dynamics by multistate IMT is also shown to have a better agreement with the all-atom nonadiabatic mapping dynamics than the Marcus theory does. Because the multistate nonlinear-response IMT is straightforward and cost-effective in implementation and accounts for the nonequilibrium nuclear effects, we believe this method offers a practical strategy for studying charge transfer dynamics in complex condensed-phase systems.

## 1 Introduction

There is a growing interest in understanding charge transfer (CT) due to its relevance with practical applications in artificial photosynthesis, optoelectronics, photocatalysis, quantum information, etc.^1−11^ In particular, photoinduced charge transfer plays a vital role in solar energy conversion, such as in organic photovoltaic (OPV) cells.^6,9,10,12−15^ In a typical photoinduced CT process, light-harvesting molecules are photoexcited from the ground state to a locally excited state, which triggers a subsequent electronic transition to a CT state. A key problem for both experimental and theoretical studies is the kinetics of the photoinduced CT processes in the condensed phase because any effective energy conversion material must be produced in the condensed phase to have a high power conversion efficiency. Ultrafast spectroscopic experimental techniques such as transient absorption^6,16−20^ and two-dimensional electronic spectroscopy^12,21−23^ have been applied to detect the signature of CT kinetics in complex condensed-phase systems. Besides ultrafast spectroscopic simulation techniques, theoretical and computational methods have proven to provide new and complementary molecular insight into CT.^24−27^

Marcus theory^28−34^ is the most widely used theory for calculating the charge transfer rate constant between two coupled electronic states and has been successfully applied to a variety of complex systems ranging from electron transfer in the gas phase^35,36^ to solutions^37^ and biological macromolecules.^38,39^ The popularity of the Marcus theory might stem from the fact that the Marcus donor-to-acceptor rate constant is given by only three parameters: the electronic coupling Γ_*DA*_, the reaction free energy Δ*E*, and the reorganization energy *E*_*r*_:
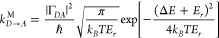
1The Marcus parabolic model offers an intuitive picture for understanding the trend of CT rate constant with an increasing thermodynamics driving force, which predicts the famous turnover from the normal regime to the inverted regime.^40−42^

Despite the enormous success, Marcus theory is based on the assumption that the initial nuclear state of the system is at thermal equilibrium on the potential energy surface (PES) of the donor (initial) state and thus cannot account for the effects caused by the initial nonequilibrium nuclear state, which are usually seen in photoinduced dynamics. For example, an extremely common photoinduced CT process starts with the initial nuclear distribution of the system at thermal equilibrium on the ground-state PES, which does not change upon the vertical photoexcitation that brings the electronic state suddenly from the ground state to the excited state.^31^ It can be shown that the instantaneous CT rate coefficient would deviate from the Marcus CT rate constant significantly for the reason that the initial nuclear distribution is different from the equilibrium distribution on the donor surface.^43,44^ Moreover, the subsequent nuclear relaxation after the vertical photoexcitation leads to a time-dependent CT rate, which may give rise to drastically different kinetics of CT compared with Marcus theory.^45−49^

Recently, we proposed the instantaneous Marcus theory (IMT),^49,50^ which is designed to account for the nonequilibrium effect of the initial nuclear state and formulated as a Marcus-like time-dependent CT rate coefficient expression with explicitly time-dependent donor–acceptor energy gap average  and its variance σ_*DA*_^2^(*t*):
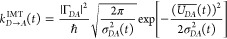
2which can also be expressed equivalently in terms of time-dependent reaction free energy Δ*E*(*t*) and reorganization energy *E*_*r*_(*t*):
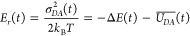
3The above IMT expression in eq 2 was derived from taking the classical limit of the nonequilibrium Fermi’s golden rule (NE-FGR),^45−55^ which provides a more accurate way based on quantum-mechanical perturbation theory to compute the time-dependent CT rate coefficient starting with arbitrary nonequilibrium nuclear initial conditions.^49^ The linearized semiclassical (LSC) approximation of NE-FGR as well as IMT opens the door to obtaining the CT rate coefficient of condensed-phase systems described by anharmonic force fields. IMT is expected to be accurate when the nuclear degrees of freedom (DOF) can be assumed classical, the time scale of nuclear motion is slower than the electronic dephasing time, and the distribution of the donor–acceptor energy gap is described by the time-dependent Gaussian form. In high-temperature condensed-phase systems, IMT can be quite accurate compared with LSC NE-FGR.^49−51^ For example, in studying the prototypical OPV carotenoid–porphyrin–C_60_ (CPC_60_) molecular triad^49,50,56−62^ dissolved in explicit tetrahydrofuran (THF) solvent at room temperature, IMT was demonstrated to reproduce the LSC NE-FGR electronic transition rate for *D* → *A* remarkably well and both approaches reveal significant nonequilibrium effects that dramatically influence the photoinduced CT dynamics.^49−51^

A great feature of IMT is that it can be implemented with classical molecular dynamics (MD) simulations to obtain the nonequilibrium average and variance of the energy gap in eq 2, making it straightforward to apply to complex systems. Directly evaluating the IMT rate in eq 2 requires nonequilibrium molecular dynamics (NEMD) simulations with initial nuclear conditions sampled from the equilibrated ground state.^49^ Although IMT requires much less computational resources than LSC NE-FGR, all-atom NEMD simulation of tens to hundreds of thousands of atoms in realistic solar energy conversion systems can still be computationally expensive. To accelerate the IMT computation, we proposed the framework of linear-response and nonlinear-response formulations of IMT that would only require equilibrium MD (EMD) simulations, which greatly reduce the computational cost compared to the original NEMD simulations.^49,50^ The underlying principle for these formulations is the fluctuation–dissipation theorem of statistical mechanics, which relates the nonequilibrium relaxation to equilibrium fluctuations such that we could use the time correlation function of fluctuations to capture the nonequilibrium effects in the IMT rate coefficient. It is worth noting that the nonlinear-response IMT formula that only requires EMD is exactly equivalent to the original IMT that depends on NEMD, and thus no further approximation is made in nonlinear-response IMT, in contrast to the linear-response IMT that invokes the classical linear-response theory. In addition, the nonlinear-response IMT does not cost more than the linear-response IMT and thus is recommended for realistic applications.

However, the previous linear-response and nonlinear-response IMT have only been applied to a certain donor-to-acceptor transition starting with a nonequilibrium initial nuclear state,^50^ but a whole picture of the photoinduced CT cannot be obtained without simulating electronic transitions between all pathways among multiple electronic states. In this work, we develop the all-atom computational strategy of IMT for simulating photoinduced CT that involves multiple excited states, which starts with the nonequilibrium initial nuclear state sampled from the ground state. The key innovation is the IMT treatment of multiple states, and the proposed multistate nonlinear-response IMT calculation only requires dynamical input from incoherent EMD simulations and generates all pairwise time-dependent rates and thus the electronic population of all states under the influence of nonequilibrium nuclear relaxation. This multistate IMT approach is believed to be practical in simulating large realistic systems described by anharmonic all-atom force fields. In particular, we will use the all-atom description of the CPC_60_ triad dissolved in explicit THF solvent and consider the following three excited states: the brightest porphyrin-localized excited ππ* state, the partially charge-separated state, CP^+^C_60_^–^ (CT1), and the fully charge-separated state, C^+^PC_60_^–^ (CT2), as well as the ground state. We hope to use the newly proposed multistate nonlinear-response IMT to simulate the simultaneous population transfer between all pairs of transitions in the three excited states after the triad was suddenly photoexcited to the ππ* state while the structural arrangement of the entire solution is still at equilibrium with the ground-state PES.

The remainder of this article is organized as follows. Sec. 2 describes the multistate nonlinear-response instantaneous Marcus theory method developed for electronic transitions between multiple states. Sec. 3 presents the two concerned different conformations of the CPC_60_ triad dissolved in explicit THF solvent. Sec. 4 describes the simulation details of the all-atom IMT calculation and all-atom nonadiabatic mapping dynamics. Sec. 5 reports and discusses the results. Sec. 6 provides the concluding remarks.

## 2 Multistate Nonlinear-Response Instantaneous Marcus Theory

In this section, we introduce the nonlinear-response IMT formulation and extend it to the general case where electronic transitions among multiple states are concerned. We consider the general *F*-state all-atom Hamiltonian that is given by
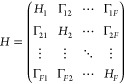
4where the interstate couplings are assumed to be constants, i.e., Γ_*jk*_ = Γ_*kj*_ = const. in the Condon case, and the nuclear Hamiltonian of the *j*th electronic state for an *N*-atom system is described by
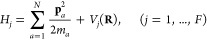
5Here, we have the atomic masses {*m*_*a*_|*a* = 1, ..., *N*}, 3*N*-dimensional nuclear coordinates **R** = (**r**_1_, ..., **r**_*N*_), and conjugate momenta **P** = (**p**_1_, ..., **p**_*N*_), as well as the PES of the *j*th state *V*_*j*_(**R**) . In the previous study,^49,50^ only a certain *D* → *A* transition is considered and the donor-state population decay as a function time is given by the following approximate exponential decay
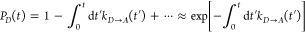
6where the time-dependent CT rate coefficient *k*_*D*→*A*_(*t*) is given by eq 2.

In this work, we consider all possible reaction pathways *j* → *k* and *k* → *j* for any different pairs of electronic states in the multistate system in eq 4. The equations of motion for the electronic population can be written in the form of Pauli’s master equations as below^63,64^
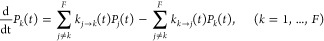
7where the time-dependent rate coefficient *k*_*j*→*k*_(*t*) for the transition *j* → *k*(*j*, *k* = 1, ..., *F*; *j* ≠ *k*) is given by IMT:
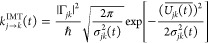
8The above expression is a generalization of eq 2 and is based on assuming that the instantaneous distribution of energy gap *U*_*jk*_(**R**) = *V*_*j*_(**R**) – *V*_*k*_(**R**) is time-dependent Gaussian:
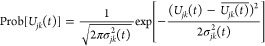
9

The IMT rate coefficient in eq 8 requires two time-dependent inputs from MD simulations including  and σ_*jk*_^2^(*t*). Here, the energy gap between the *j*th and the *k*th PESs is a function of the configuration that evolves with time (we use the simplified notation here for a dynamical variable that is a function of phase space point at a given time):
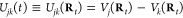
10and the overbar indicates the nonequilibrium averages with initial conditions sampled from the classical ground state equilibrium distribution ρ_*g*_(**R**_0_, **P**_0_) on ground-state surface *V*_*g*_(**R**) . Direct evaluation of the nonequilibrium average would require NEMD, which can be computationally demanding in large condensed phases. Besides, we can also compute the effective time-dependent activation energy from the energy gap and its variance such that the exponential term in eq 8 becomes exp[−*E*_*a*_(*t*)/*k*_*B*_*T*]:
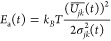
11

Next, we describe the multistate nonlinear-response IMT formulation based on EMD that is equivalent to but more cost-effective than the direct evaluation using NEMD. First,  is the nonequilibrium average of energy gap *U*_*jk*_(**R**) = *V*_*j*_(**R**) – *V*_*k*_(**R**), which is obtained with starting from the initial state (**R**_0_, **P**_0_) sampled from the equilibrated ground state, and propagated on the PES of the initial electronic state *V*_*j*_ for time *t* and arrives at (**R**_*t*_, **P**_*t*_), leading to instantaneous *U*_*jk*_(*t*) = *U*_*jk*_(**R**_*t*_). The average of *U*_*jk*_(*t*) over many trajectories generates . To be specific, the nonequilibrium average of the energy gap is defined as^50^
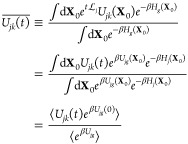
12where **X**_0_ = (**R**_0_, **P**_0_) is the initial phase-space point,  is the classical Liouville operator that propagates the system according to the *j*th state Hamiltonian *H*_*j*_ as in , and ⟨•⟩ is the equilibrium average of the *j*th state. If we define *δA* = *A* – ⟨*A*⟩ as the fluctuations of any dynamical variable *A* about its equilibrium average, eq 12 can be written as
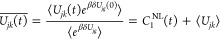
13where the first-kind nonlinear-response IMT time correlation function (TCF) is given by
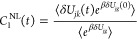
14

Second, the nonequilibrium variance of energy gap *U*_*jk*_ at time *t* after the photoexcitation is defined as below
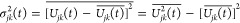
15where
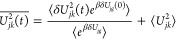
16with
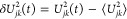
17Inserting eq 13 and eq 16 into eq 15, we have the nonlinear-response expression for the variance of the energy gap:
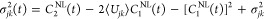
18where
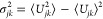
19and the second-kind nonlinear-response IMT TCF is given by
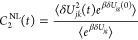
20

Thus, eq 13 and eq 18 are the nonlinear-response prescriptions for the time-dependent average and variance for energy gap *U*_*jk*_ for transition *j* → *k*. Now that  and σ_*jk*_^2^(*t*) are written in terms of TCF and statistical averages, one can evaluate them by using EMD simulations, taking advantage of time translational symmetry to boost the sampling efficiency. In the practice of multistate IMT, one needs to run one or several independent long equilibrium trajectories on the initial electronic state’s PES (*V*_*j*_) and recalculate the corresponding energy gaps (*U*_*jk*_ for transition *j* → *k*). This means that the nuclear dynamics sampled on the initial electronic state’s PES for different transition pathways will be different from different initial electronic states, and it only reflects the nonequilibrium relaxation following vertical excitation from the ground state to the particular initial electronic state; thus, the population dynamics is estimated incoherently. For all combinations of reaction directions, EMD simulations have to be executed on the initial electronic state for all possible transitions. It is noted that the nonlinear-response IMT formula for each transition is numerically exact and equivalent to the direct definition of nonequilibrium classical statistical mechanics.

## 3 CPC_60_ Triad in Explicit THF Solvent

**Figure 1:**
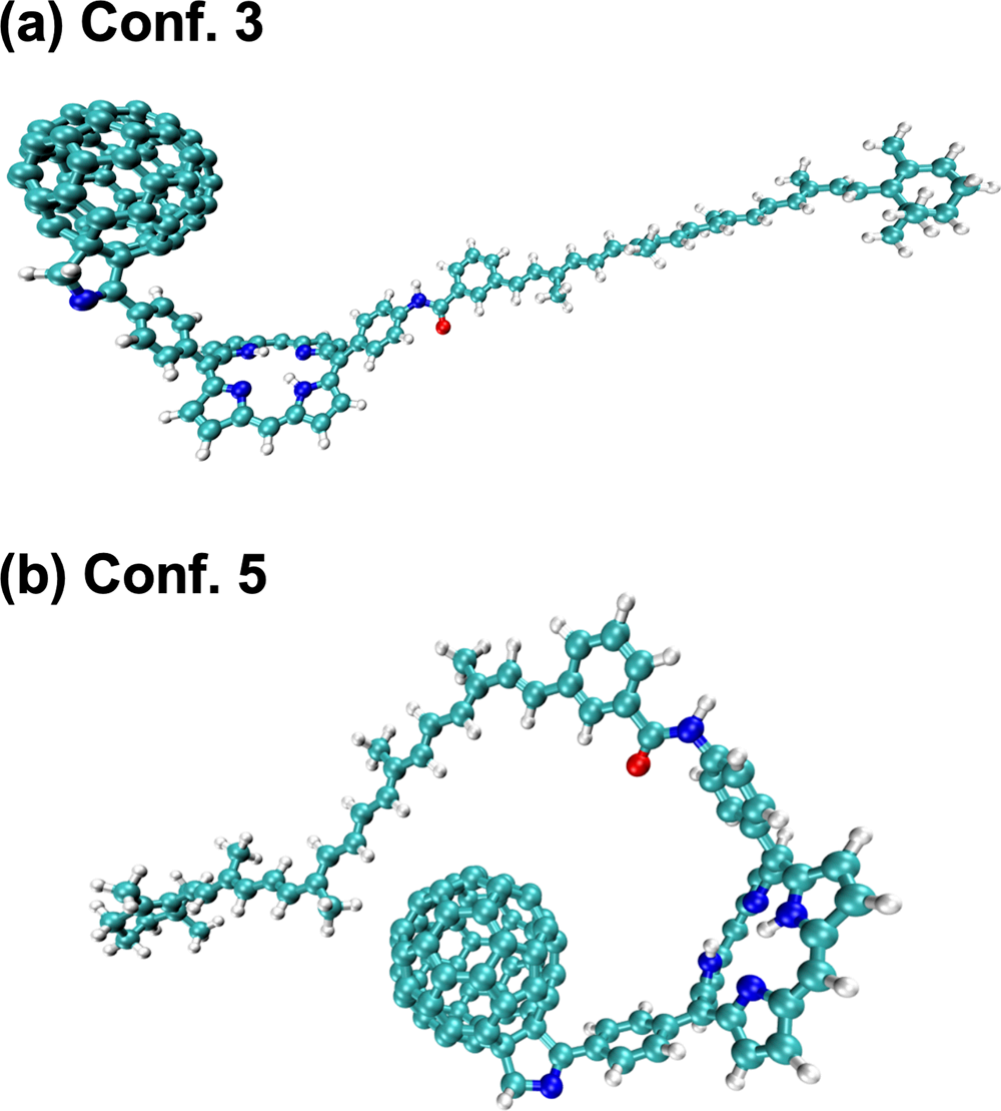
Geometries of triad conformations 3 (a) and 5 (b).

In this work, we employed the prototypical OPV CPC_60_ triad dissolved in explicit THF solvent to serve as a proof of principle for the multistate nonlinear-response IMT calculation in condensed-phase systems. All-atom multistate Hamiltonians were constructed for two triad conformations, i.e., Conf. 3^65^ and Conf. 5^66^ shown in Figure 1, which were selected from the landmark structure database^67^ to exhibit population transfer dynamics among all three excited states in the first few picoseconds after the photoexcitation. For each conformation, four electronic states including ππ*, CT1, CT2, and ground state (g) were parametrized. All-atom force fields for the CPC_60_ triad in explicit THF were modified from the general Amber force field (GAFF) potential,^68^ which differ concerning the atomic partial charge and the excitation energies of the triad.^50,62,69^ Electronic structure calculation of the triad molecule was performed on the level of time-dependent density functional theory under Tamm–Dancoff approximation (TDA-DFT)^70^ with splitting valence (SV) basis set using range-separated hybrid (RSH) Baer–Neuhauser–Livshits (BNL) functional^71^ with QChem 4.4 package,^72^ which was used to determine the atomic partial charges from Mulliken charges, the excitation energies, and the electronic couplings for the triad. In the BNL functional, system-dependent range separation parameter ω = 0.157 was used for the triad molecule in the gas phase. The interstate electronic couplings were calculated by the fragment charge difference (FCD) method^73^ via TDA-DFT with the BNL functional and SV basis set. For the THF solvent, the GAFF parameters adopted the restrained electrostatic potential (RESP) atomic partial charges calculated at the B3LYP/6-311G* level using the Gaussian 16 package.^74^

The all-atom MD simulation box consists of one CPC_60_ triad dissolved in 2700 THF solvent molecules (a total of 35,307 atoms) in a 70 Å × 70 Å × 70 Å box with periodic boundary conditions. In most MD field fields, the reference zero of total potential energy is chosen arbitrarily since it does not affect the dynamics or the gradients (forces) on a single potential energy surface. However, when multiple electronic PESs are concerned, we will have to consider not only the gradients (forces) but also the relative vertical energy shifts between different electronic states. The main effect of the vertical shifts comes from the excitation energy of the solute molecule, i.e., the triad, while it is embedded in the solvent environment. Thus, the total potential energies of different electronic states need to account for the excitation energy of the triad as well as the interaction with the solvent. To this end, we adopted the strategy from refs (49, 62), and (69) and used the following total potential energy expression for *j*th state:
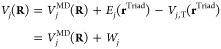
where *V*_*j*_^MD^(**R**) is the force field potential energy of the *j*th state triad soaked in explicit THF, *E*_*j*_(**r**^Triad^) is the excitation energy of the triad on the *j*th state in the gas phase via quantum chemistry calculation, *V*_*j*,T_(**r**^Triad^) is the force field potential energy of the *j*th state triad in the gas phase, which is removed to avoid double-counted energy of triad itself that has already included in *E*_*j*_(**r**^Triad^), and thus *W*_*j*_ serves as the energy correction to the force field potential energy. Here, **R** denotes the positions of the solute triad and the solvent molecules and **r**^Triad^ denotes the gas-phase triad geometry. Table 1 summarizes the essential energetic parameters of the two triad conformations.

**Table 1:** Energy Minima ε_*j*_ and Energy Corrections *W*_*j*_ of Four Electronic States and Electronic Couplings Γ_*jk*_ and Reorganization Energies *E*_*r*_^(*jk*)^ between Different Pairs of States in Two Conformations (3 and 5) of the Triad (Energy Unit in eV except for *E*_*r*_^(*jk*)^ in kcal/mol)^a^

	Conf. 3	Conf. 5
ε_1_(ππ*)	0	0
ε_2_(CT1)	–0.828	–0.758
ε_3_(CT2)	–0.640	–1.128
ε_4_(g)	0	0
*W*_1_(ππ*)	0.728	1.551
*W*_2_(CT1)	–2.103	–0.697
*W*_3_(CT2)	–2.131	–0.650
*W*_4_(g)	0	0
Γ_12_	–1.5 × 10^–2^	8.1 × 10^–2^
Γ_13_	7.2 × 10^–3^	4.1 × 10^–3^
Γ_23_	–2.9 × 10^–2^	–3.2 × 10^–3^
Γ_*j4*_	0	0
*E*_*r*_^(12)^	7.880	6.464
*E*_*r*_^(13)^	11.39	18.68
*E*_*r*_^(14)^	0.9202	20.22
*E*_*r*_^(23)^	3.546	0.3096
*E*_*r*_^(24)^	21.23	7.920
*E*_*r*_^(34)^	26.42	18.95

a Here, electronic states *j*, *k* = 1, 2, 3, 4 correspond to ππ*, CT1, CT2, and ground (g) states, respectively. The electronic couplings between any excited states (*j* < 4) and the ground state are assumed to be zero.

## 4 Simulation Details

### 4.1 All-Atom Nonlinear-Response IMT

The multistate nonlinear-response IMT calculation only requires equilibrium MD simulations on the initial electronic state’s PES. For example, for transition *j* → *k*, the equilibrium trajectory on the *V*_*j*_ PES is required for calculating the time correlation functions *C*_1_^NL^(*t*) and *C*_2_^NL^(*t*) in eqs 14 and 20, respectively. All-atom MD simulations were performed using the PMEMD program of the AMBER22 package.^75^ The particle mesh Ewald (PME) algorithm is utilized to compute the electrostatic interaction.^76^ The nonbonded interaction is cutoff at 9 Å. The covalent bonds evolving hydrogen atoms are constrained by the SHAKE algorithm,^77^ and the atoms in the triad molecule are restrained by the harmonic force constant 100 kcal mol^–1^. The MD time step is 1 fs in this work, if not mentioned explicitly.

To prepare the systems of the two triad conformations, we first performed energy minimization using the steepest gradient approach for the first 1000 steps followed by 39000 conjugate gradient minimization with an initial step length of 0.01 ps. The system was then heated to 300 K for 20 ps and relaxed for 80 ps at 300 K using a Langevin thermostat with a friction coefficient of 2.0 ps^–1^ with a time step Δ*t* = 2 fs. The system is equilibrated under the NPT ensemble at 300 K and a pressure of 1 bar for 0.5 ns using an anisotropic Berendsen barostat with a pressure relaxation time of 0.5 ps and compressibility of 4.46 × 10^–5^ bar and Langevin thermostat with a friction coefficient of 1.0 ps^–1^. From the NPT simulations, the equilibrium box size for triad Conf. 3 is 71.6 Å × 71.6 Å × 71.5 Å and equilibrium box size for triad Conf. 5 is 71.5 Å × 71.6 Å × 71.5 Å), which will be used for subsequent constant-volume simulations. The system was switched to the NVT ensemble at 300 K with a Langevin thermostat with a friction coefficient of 1.0 ps^–1^ for equilibration of 500 ps, and then 1000 independent canonical initial conditions were sampled every 50 ps for Conf. 3. For Conf. 5, the first 200 initial conditions were sampled every 20 ps, and then the rest were sampled every 50 ps. For both Conformations, 1000 independent NVE trajectories starting with the canonically sampled initial conditions were propagated for 100 ps under the NVE ensemble after re-equilibration of 50 ps, and snapshots of the configurations were recorded every 5 fs for time correlation functions *C*_1_^NL^(*t*) and *C*_2_^NL^(*t*) calculation whose statistical error bars were computed by splitting the 1000 trajectories into 4 batches.

### 4.2 All-Atom Nonadiabatic Mapping Dynamics

The all-atom nonadiabatic dynamics for the triad in THF solvent were obtained by using the symmetrical quasiclassical (SQC) and the linearized semiclassical (LSC) methods.^69,78−81^ Both methods are based on the Meyer–Miller–Stock–Thoss mapping Hamiltonian^82,83^ and have been shown to give accurate nonadiabatic dynamics for condensed phase systems. Data of the nonadiabatic dynamics of SQC and LSC for triad Conf. 3 were obtained from ref (69). The nonadiabatic dynamics results of Conf. 5 newly reported here were simulated with the QCDyn program with GPU acceleration using modified OpenMM 7.5.0.^84^ The PME algorithm was utilized to compute the electrostatic interaction;^85^ the van der Waals interaction cutoff distance is 9.0 Å, and the dispersion correction for long-range van der Waals interaction is applied. The bonds involving hydrogen atoms are constrained, and the atoms in triad molecule are restrained by a harmonic force of 100 kJ mol^–1^ Å^–2^. The nuclear time step Δ*t* = 1 fs.

To prepare the system of triad Conf. 5 in explicit THF solvent, we first perform an energy minimization of up to 10000 iterations or the root-mean-square of all force components including bonding and nonbonding components reaches 10 kJ mol^–1^ nm^–1^ on ground state PES. The system was then heated up from 0 to 300 K increasing 1 K per 333 fs on ground state PES. The system is equilibrated under the NPT ensemble for 1 ns using a Monte-Carlo barostat^86,87^ per 25 steps at 1 bar and Langevin thermostat with a friction coefficient of 1.0 ps^–1^, which resulted in an averaged box size of 71.6 Å × 71.7 Å × 71.6 Å. The system is equilibrated under the NVT ensemble for 1 ns using a Langevin thermostat with a friction coefficient of 1.0 ps^–1^, which is then used to sample independent initial canonical conditions of position and velocities every 1 ns. Finally, the nonadiabatic dynamics were obtained by averaging over 10^4^ independent nonadiabatic trajectories starting with the ππ* population and the sampled nuclear initial conditions from the equilibrated ground state. We refer the reader to ref (69) for more details of all-atom nonadiabatic dynamics methods. The electronic mapping variables were propagated 20 times by the fourth-order Runge–Kutta algorithm in one nuclear step. The zero-point-energy (ZPE) parameter was chosen as γ = 1/2 in the linearized semiclassical mapping type 1 and 2 (LSC1 and LSC2) dynamics, corresponding to Poisson bracket mapping equation (PBME)^81^ and LSC initial value representation (LSC-IVR),^80^ respectively,^69,80,81,88,89^ and γ = 1/3 in symmetrical quasiclassical method with triangle window function.^69,90^

## 5 Results and Discussion

**Figure 2:**
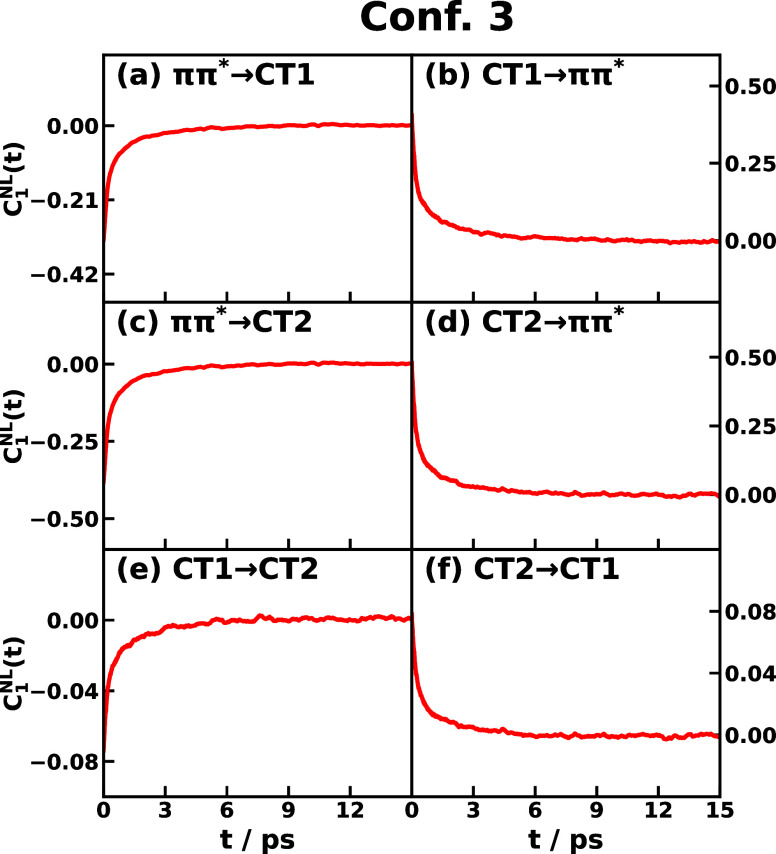
Time correlation function *C*_1_^NL^(*t*) of CPC_60_ triad conformation 3 for different transitions *j* → *k* obtained from all-atom equilibrium MD simulations on the *V*_*j*_ potential surface at 300 K.

We first present the time correlation functions *C*_1_^NL^(*t*) for triad Conf. 3 and Conf. 5 in Figures 2 and S3, respectively. A total of six transitions among the three excited states, including ππ* ↔ CT1, ππ* ↔ CT2, and CT1 ↔ CT2, are considered, where each pair of electronic states has corresponding forward and backward reaction directions. An obvious feature is that *C*_1_^NL^(*t*) approaches zero in the long-time limit, which is expected since the time-*t* observable is a fluctuation, for example, the energy gap fluctuation *δU*_*jk*_(*t*) is in the numerator of *C*_1_^NL^(*t*), i.e., ⟨*δU*_*jk*_(*t*)*e*^*βδU*_*jg*_(0)^⟩ and the denominator ⟨*e*^*βδU*_*jg*_^⟩ is always positive. The time scale for the TCF to relax is about 6 ps for most of the transitions in Confs. 3 and 5. Another observation is that the forward and backward transitions seem to have opposite signs in short-time *C*_1_^NL^(*t*), with only one exception in Conf. 5 in Figure S3e,f. In fact, the signs of *C*_1_^NL^(*t*) for forward and backward transitions are not always anticorrelated, since the TCF is a function of fluctuations of energy gaps instead of energy gaps themselves.

To have a better picture, considering forward and backward reactions *j* ↔ *k*, the corresponding numerator TCFs of *C*_1_^NL^(*t*) are
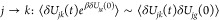
21
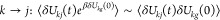
22where the linear TCFs such as ⟨*δU*_*jk*_(*t*)*δU*_*jg*_(0)⟩ would dominate the exponential TCFs. In the case of opposite signs such as in ππ* ↔ CT1 reactions of Conf. 3 in Figure 2a,b, that *δU*_*jk*_(*t*) anticorrelates with *δU*_*jg*_(0) giving rise to negative TCF, whereas *δU*_*kj*_(*t*) correlates with *δU*_*kg*_(0) giving rise to positive TCF, which is consistent with the picture that *j* fluctuates in between *k* and *g* at some instances of sampling time as shown in Figure S1a. In the case of the same sign such as CT1 ↔ CT2 reactions of Conf. 5 in Figure S3e,f, the two energy gap fluctuations can be both positive or both negative, leading to positive TCFs for the forward and backward reactions, which is consistent with the picture that *g* fluctuates in between *j* and *k* as shown in Figure S1b. So the TCF *C*_1_^NL^(*t*) depending on the conformation, the transition, and the sampling PES could be positive or negative in the short time and approach zero in the long time.

**Figure 3:**
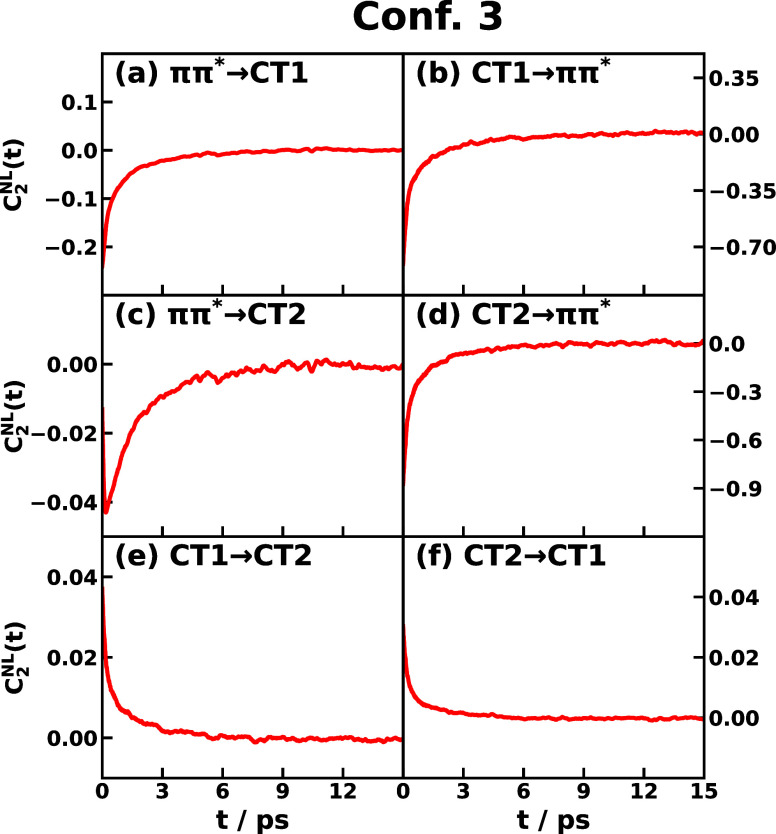
Time correlation function *C*_2_^NL^(*t*) of CPC_60_ triad conformation 3 for different transitions *j* → *k* obtained from all-atom equilibrium MD simulations on the *V*_*j*_ potential surface at 300 K.

Next, we present the time correlation functions *C*_2_^NL^(*t*) for triad Conf. 3 and Conf. 5 in Figures 3 and S4, respectively. The six transitions among the three excited states of ππ*, CT1, and CT2 are plotted. Besides the feature of approaching zero in about 6 ps in all the transitions, we observe that the signs of TCF *C*_2_^NL^(*t*) of forward and backward reactions tend to be the same, which could be attributed to the time-*t* fluctuation of *δU*_*jk*_^2^(*t*) that reflects the relative separating speed of *V*_*j*_ and *V*_*k*_ from the equilibrium value. As shown in Figure S2a, when the fluctuation *δU*_*jk*_^2^(*t*) > 0 we have *j* and *k* fluctuate to move away from each other, say *j* moves upward and *k* moves downward, if the fluctuation of *g* energy exceeds the speed of *j* moving upward, then we would have *δU*_*jg*_(0) < 0 and *δU*_*kg*_(0) < 0, leading to negative *C*_2_^NL^(*t*) for both *j* → *k* and *k* → *j* directions, which is seen in most of the cases. Similarly is shown in Figure S2b that when the fluctuation of *g* energy exceeds the speed of *k* moving downward, we would have *δU*_*jg*_(0) > 0 and *δU*_*kg*_(0) > 0, leading to postive *C*_2_^NL^(*t*) for both *j* → *k* and *k* → *j* directions, which is seen in Conf. 3 CT1 ↔ CT2 cases in Figure 3e,f. We stress that the realistic fluctuations that contribute to the final TCFs would be more complicated than the simple schematic illustrations shown in Figure 3. To ensure the *C*_1_^NL^(*t*) and *C*_2_^NL^(*t*) have converged with all-atom MD simulations, we show the error bars in Figures S11 and S12 for Conf. 3 and Figures S14 and S15 for Conf. 5, respectively.

**Figure 4:**
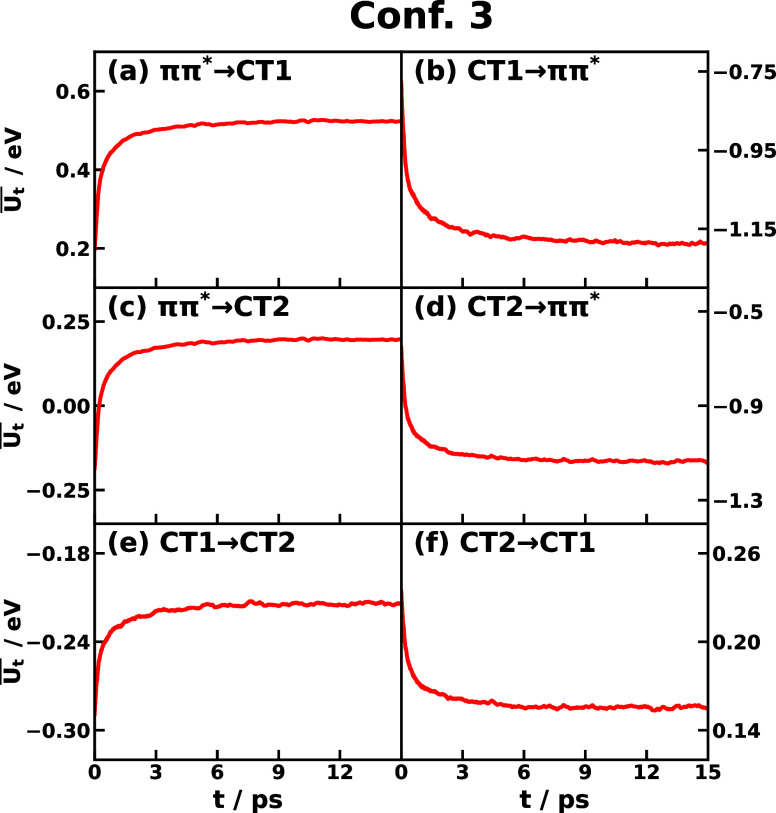
Time-depenent average energy gap  of CPC_60_ triad conformation 3 for different transitions *j* → *k* obtained from all-atom equilibrium MD simulations on the *V*_*j*_ potential surface at 300 K.

The time-dependent energy gap average  can be obtained by using eq 13 with the input of *C*_1_^NL^(*t*) and the ensemble average of ⟨*U*_*jk*_⟩ from Table 2. The time-dependent energy gap averages of Conf. 3 and Conf. 5 are offered in Figures 4 and S5, respectively, which have the same time-dependent trend as the *C*_1_^NL^(*t*) as expected. The absolute change in the energy gap average can be significant, for example,  takes the value of −0.4 eV at time zero to a plateau value of −1.1 eV at 15 ps in Conf. 3 CT2 → ππ* shown in Figure 4d. The typical nuclear relaxation time scale is several picoseconds, such as 4 to 6 ps for Conf. 3 shown in Figure 4. The time evolution of the energy gap reflects the nonequilibrium effect due to the initial nuclear preparation on the ground state, and the amount of the energy-gap change is related to how far the initial electronic state of the transition is away from the equilibrium position on the ground electronic state. A quantitative description of the distances between different electronic states could be achieved by mapping the all-atom Hamiltonian to the multistate reaction coordinate (MRC) model Hamiltonian.^66^

**Table 2:** Ensemble Average ⟨*U*⟩ (eV) and Variance σ^2^ (eV^2^) of the Energy Gap, Reorganization Energy *E*_*r*_(eV), Marcus Rate Constant *k*^M^(s^–1^), and the Plateau Value of the IMT Rate Coefficient *k*^plat^ (s^–1^) for Different Transitions in Triad Conformations 3 and 5

	ππ* → CT1	ππ* → CT2	CT1 → ππ*	CT1 → CT2	CT2 → ππ*	CT2 → CT1
Conf. 3						
⟨*U*⟩	0.5224	0.1951	–1.184	–0.2151	–1.134	0.1562
σ^2^	0.01702	0.02471	0.01547	0.001568	0.02119	0.001314
*E*_*r*_	0.3293	0.4779	0.2993	0.03033	0.4099	0.02542
*k*^M^	5.016 × 10^8^	9.382 × 10^10^	1.664 × 10^–7^	8.010 × 10^6^	4.034 × 10^–2^	1.714 × 10^9^
*k*^plat^	1.551 × 10^9^	5.699 × 10^11^	8.078 × 10^–8^	2.647 × 10^7^	1.133 × 10^–2^	6.595 × 10^9^
Conf. 5						
⟨*U*⟩	0.4841	0.3207	–1.0324	–0.5240	–1.965	–1.314
σ^2^	0.01332	0.04115	0.01270	0.04269	0.03988	0.04554
*E*_*r*_	0.2576	0.7958	0.2456	0.8257	0.7713	0.8807
*k*^M^	8.696 × 10^9^	1.474 × 10^10^	2.036 × 10^–4^	1.346 × 10^9^	1.941 × 10^–10^	3.124 × 10^2^
*k*^plat^	3.135 × 10^10^	9.017 × 10^10^	1.606 × 10^–5^	6.988 × 10^9^	1.227 × 10^–10^	5.372 × 10^2^

**Figure 5:**
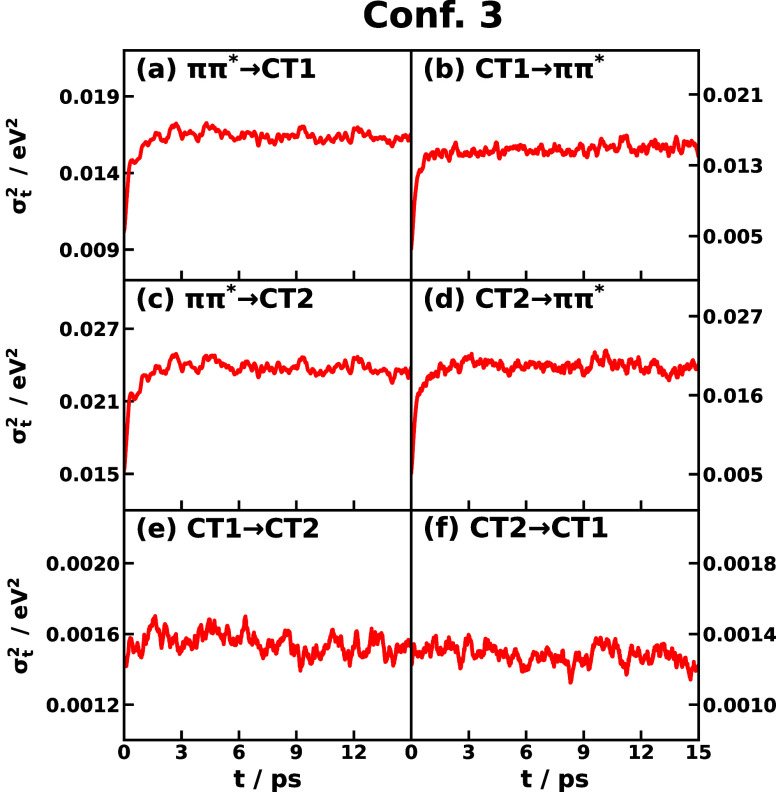
Time-dependent energy gap variance  of CPC_60_ triad conformation 3 for different transitions *j* → *k* obtained from all-atom equilibrium MD simulations on the *V*_*j*_ potential surface at 300 K.

**Figure 6:**
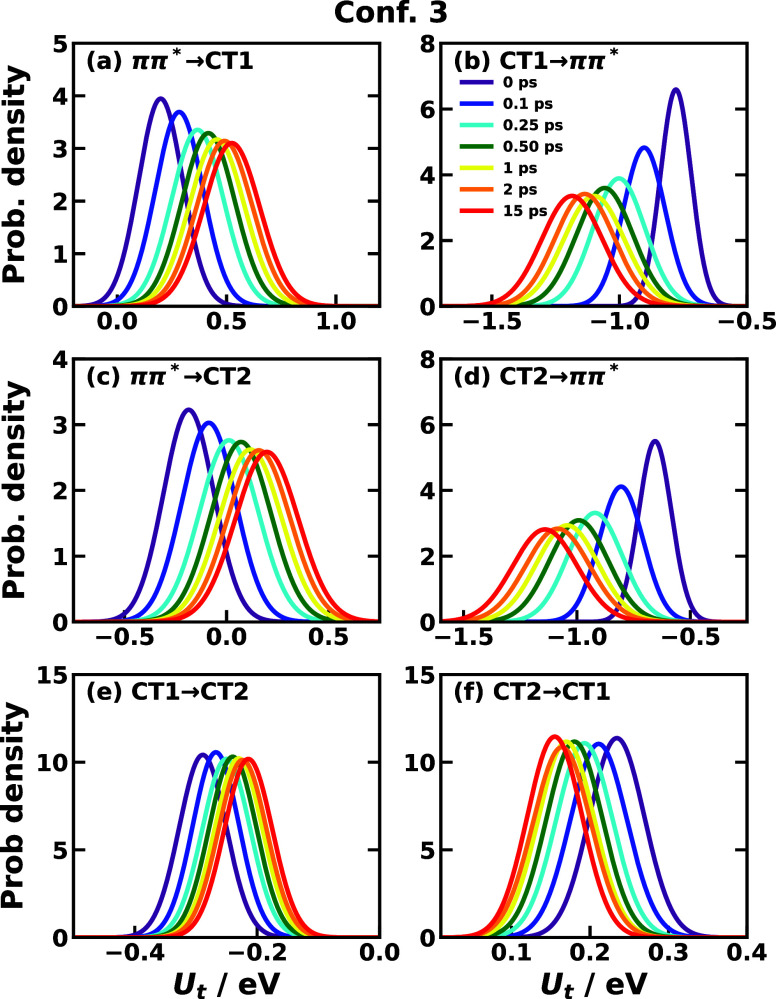
Time-dependent distribution of *U*_*jk*_(*t*) of CPC_60_ triad conformation 3 for different transitions *j* → *k* obtained from all-atom equilibrium MD simulations on the *V*_*j*_ potential surface at 300 K.

**Figure 7:**
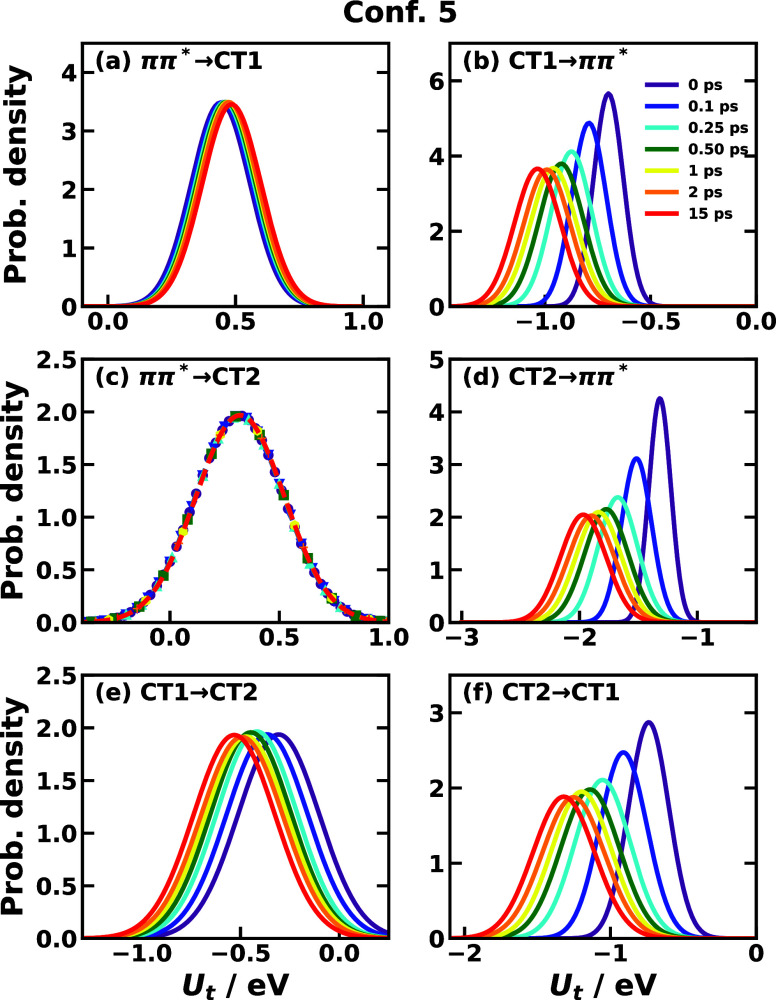
Time-dependent distribution of *U*_*jk*_(*t*) of CPC_60_ triad conformation 5 for different transitions *j* → *k* obtained from all-atom equilibrium MD simulations on the *V*_*j*_ potential surface at 300 K.

The time-dependent energy gap variance σ_*jk*_^2^(*t*) is obtained with eq 18, and the results of Confs. 3 and 5 are presented in Figures 5 and S6, respectively. The equilibrium energy-gap variances σ_*jk*_^2^ are given in Table 2. Unlike the average value that has a slower relaxation, the variance of the energy gap shows a sharp increase in the first 2 ps, and then, the variance stays stable onward. The time dependence of the energy-gap variance differentiates all-atom IMT from the harmonic models, where the variance of the energy gap is constant throughout the entire process in harmonic models.^91^ Combining the time-dependent average and variance of the energy gap, we display the time-dependent Gaussian distribution for the energy gap in Figures 6 and 7 for Confs. 3 and 5, respectively, which directly exhibit the time-dependent feature of IMT. In most cases, the energy-gap distribution displays a clear trend of shifting as a function of time, and the shifting directions are usually complementary as the average energy gap except for Conf. 5’s CT1 ↔ CT2 reactions (Figure 7e,f). Also, the fact that the energy gap distribution does not change much in Conf. 5’s transitions ππ* → CT1 and ππ* → CT2 (Figure 7a,c) suggests that the nonequilibrium effect for transitions starting from the ππ* state with initial nuclear DOF sampled from the ground state in Conf. 5 is not significant. In other words, the nuclear distributions of the ground state and the ππ* state are similar but not identical (a slight change is observed in the ππ* → CT1 transition). The width of the distribution is observed to increase, which can be traced back to the fact that σ_*jk*_^2^(*t*) usually increases after photoexcitation shown in Figures 5 and S6. The increasing spread of energy-gap distribution implies that the equilibrium distribution on the ground state is more focused, and after photoexcitation bringing to the ππ* PES, the nonequilibrium distribution gets wider and thus could access more configurational region than on the ground state.

**Figure 8:**
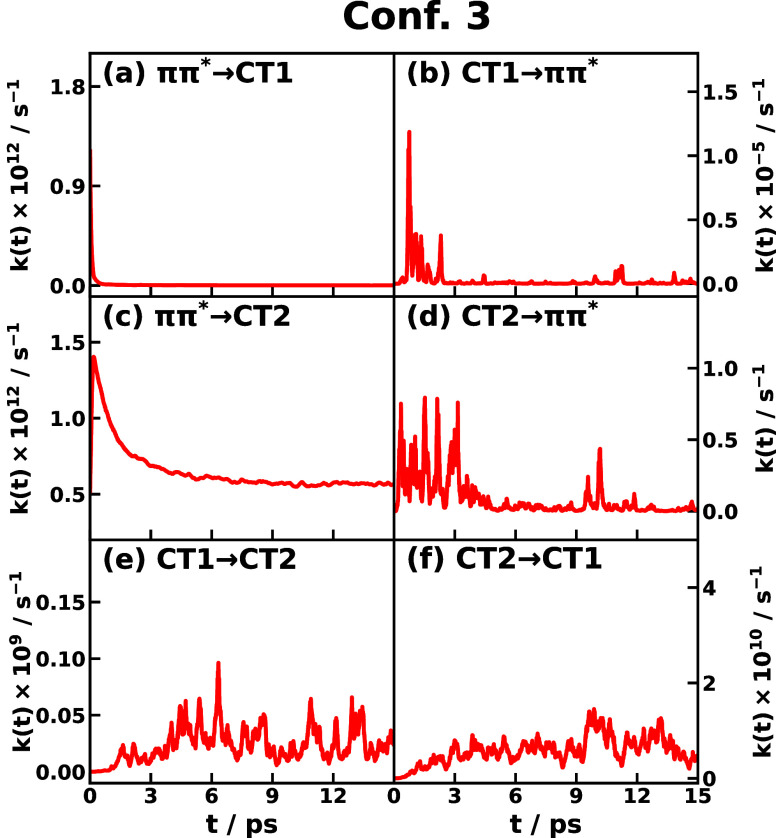
Instantaneous Marcus theory (IMT) CT rate coefficient *k*_*j*→*k*_(*t*) of different transitions *j* → *k* of CPC_60_ triad conformation 3 dissolved in THF solvent at 300 K obtained using the nonlinear-response formulation of IMT.

The time-dependent IMT rate coefficients can then be calculated by using the energy-gap average  and variance σ_*jk*_^2^(*t*). From the IMT rates of triad Conf. 3 shown in Figure 8, we recognize the large span of the order of magnitude in the IMT rate in different transitions ranging from 1 Hz to 10^12^ Hz. The largest IMT rate is observed in the transient ππ* → CT1 process, which starts with about 2 × 10^12^ Hz and decays to 1.5 × 10^9^ Hz in 0.5 ps. A comparable large IMT rate is found in the ππ* → CT2 process, which starts with about 1.4 × 10^12^ Hz and slowly decays to 6 × 10^11^ Hz in about 5 ps. The relaxation time scale for ππ* → CT2 is much slower than for ππ* → CT1, which could be ascribed to the nonequilibrium nuclear distribution moving away from the ππ*/CT1 crossing region more rapidly than from the ππ*/CT2 crossing region. The backward reaction IMT rates of CT1 → ππ* and CT2 → ππ* are negligible compared with the forward reaction rates. For the reason that the IMT rate of ππ* → CT2 maintains at the THz level for a longer time than that of ππ* → CT1, we expect that CT2 would be more populated than CT1, at least for some time before a much longer time scale dynamics becomes dominant. Of course, one also needs to consider the exchange rates between CT1 and CT2, where CT1 → CT2 is about 10^8^ Hz and CT2 → CT1 is about 10^10^ Hz; these are about 2 orders of magnitude smaller than coming from the ππ* state.

**Figure 9:**
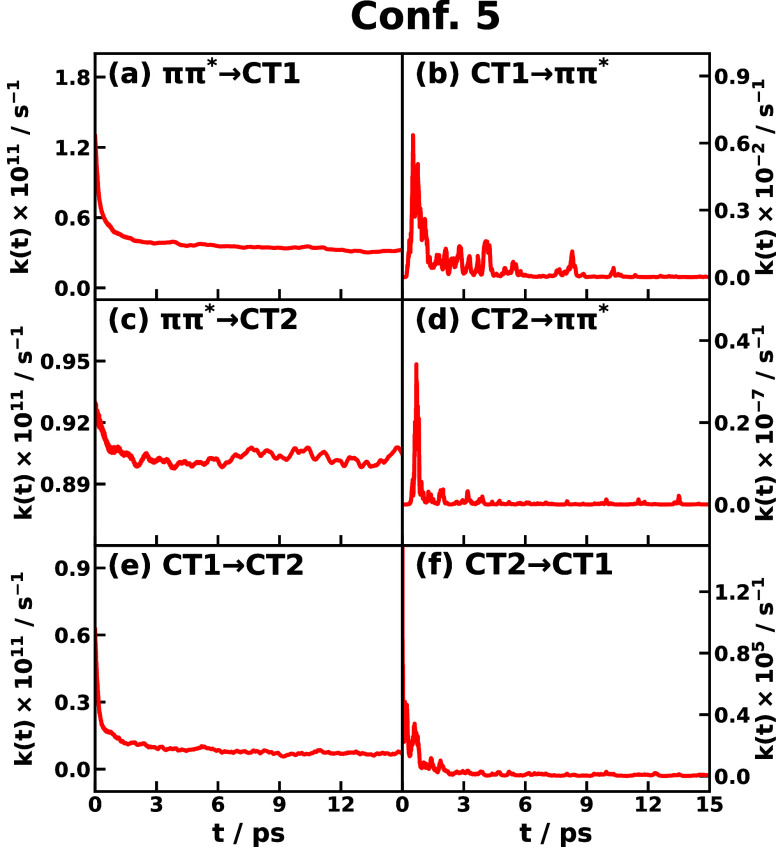
Instantaneous Marcus theory (IMT) CT rate coefficient *k*_*j*→*k*_(*t*) of different transitions *j* → *k* of CPC_60_ triad conformation 5 dissolved in THF solvent at 300 K obtained using the nonlinear-response formulation of IMT.

The IMT rates of triad Conf. 5 are shown in Figure 9, which spans from 10^–7^ to 10^11^ Hz. The largest IMT rates are seen in transitions ππ* → CT1, ππ* → CT2, and CT1 → CT2, which are on a similar level of about 10^11^ Hz and have a similar decay time scale of about 3 ps. On the other hand, the corresponding reverse transitions are completely negligible. The plateau value of the IMT rate of ππ* → CT2 is about 9 × 10^10^ Hz, which is three times as large as that of ππ* → CT1 and 13 times as large as that of CT1 → CT2. The plateau values of IMT rates of both Confs. 3 and 5 are given in Table 2 along with the equilibrium Marcus rate constants, which suggests that the time-dependent IMT rate coefficients would approach the Marcus rate constants in the long time limit, and the plateau values are within 1 order of magnitude region of the Marcus rate constant. In addition, the effective activation energy *E*_*a*_(*t*) for each transition computed from the energy-gap average  and variance σ_*jk*_^2^(*t*) using eq 11 show an expected anticorrelation with the time-dependent rate as shown in Figures S9 and S10 for Confs. 3 and 5, respectively. It can be seen that the time dependence of both the rate and the activation energy is primarily ascribed to the time dependence of the energy gap.

**Figure 10:**
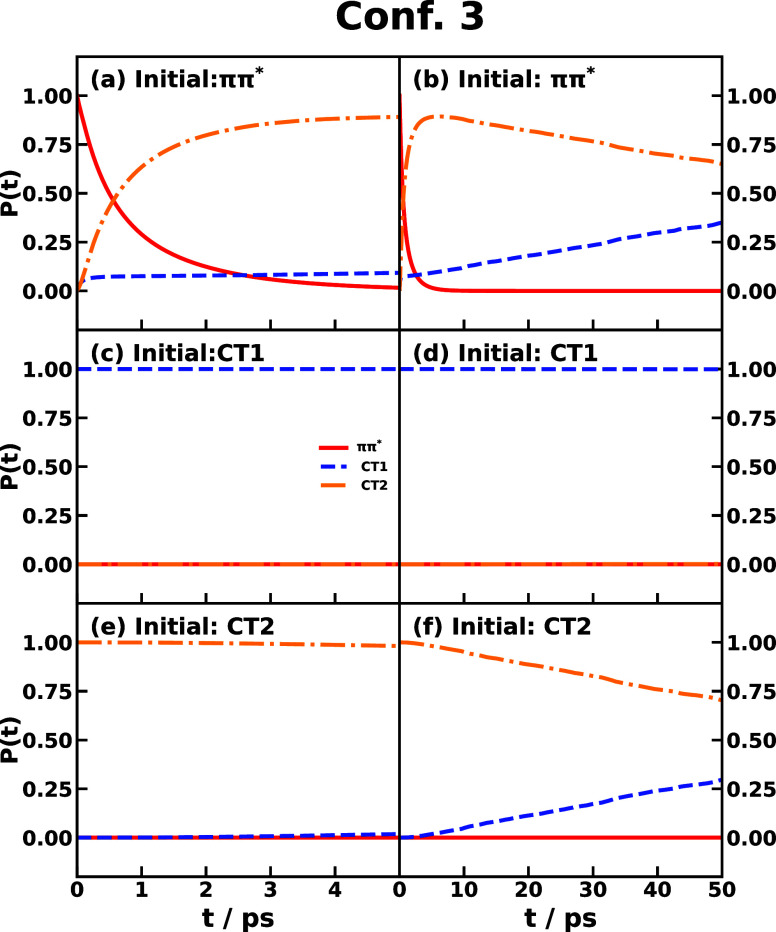
Population dynamics of CPC_60_ triad conformation 3 obtained by integrating the time-dependent IMT rate coefficient. The top, middle, and bottom panels correspond to the initial electronic states of ππ*, CT1, and CT2, respectively. In all cases, the initial nuclear state is in thermal equilibrium with the ground-state potential surface. Left and right panels are short and long-time dynamics, respectively.

Once the time-dependent IMT rates are obtained for all forward and backward transitions, we could compute the electronic population dynamics starting with a population in any state, since the IMT rates are obtained incoherently and will not be affected by which is the initially populated state. We display the population dynamics in triad Conf. 3 starting from the initial population on ππ*, CT1, or CT2 states and the initial nuclear distribution on the ground state in Figure 10. The normal photoinduced CT process would start with the ππ* population as a result of photoexcitation from the ground state to the bright ππ* state. The IMT result starting with the ππ* population is shown in Figure 10a,b, where we see the population of ππ* transfers to CT2 in the first 5 ps, and then the population of CT1 starts to rise after 10 ps. This is as expected from the IMT rates since ππ* → CT2 is kept at the THz level for a longer time than ππ* → CT1 and the CT2 → CT1 transition is faster than the reverse transition. If starting with CT1 population, there will not be any population transfer as shown in Figure 10c,d, suggesting that CT1 is the final state after a thermodynamically long time. This can be confirmed by starting with the CT2 population, which shows a slow transfer from CT2 to CT1 in the 50 ps time scale.

**Figure 11:**
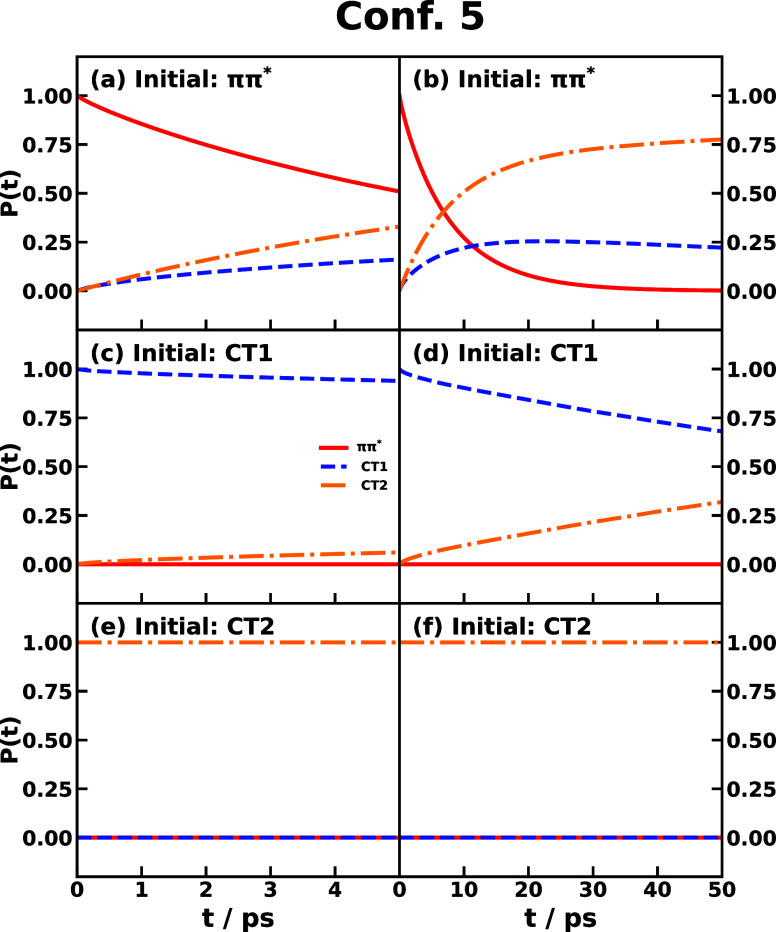
Population dynamics of CPC_60_ triad conformation 5 obtained by integrating the time-dependent IMT rate coefficient. The top, middle, and bottom panels correspond to the initial electronic state of ππ*, CT1, and CT2, respectively. In all cases, the initial nuclear state is in thermal equilibrium with the ground-state potential surface. Left and right panels are short- and long-time dynamics, respectively.

Figure 11 exhibits the population dynamics in triad Conf. 5 starting from the initial population on ππ*, CT1, or CT2 states and the initial nuclear distribution according to the ground state PES. The normal photoinduced CT starting from ππ* population produces a transfer to CT2 state more than to CT1 state for two collaborative factors: one is that ππ* → CT2 is the largest rate, and the other is that CT1 → CT2 is much faster than the reverse reaction. Thus, the CT2 state is the final populated state after a thermodynamically long time, which can be confirmed from the behavior that CT2 would be populated in 50 ps when starting from the CT1 population, and no population transfer will occur when starting from the CT2 population.

**Figure 12:**
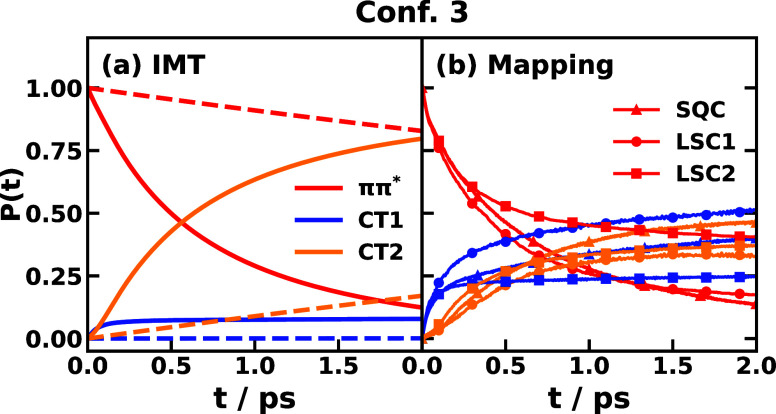
Comparison of population dynamics of CPC_60_ triad conformation 3 dissolved in explicit THF solvent at 300 K with the initial electronic state on ππ* obtained with (a) instantaneous Marcus theory (IMT, solid) and traditional Marcus theory (dashed) and (b) nonadiabatic mapping dynamics using the symmetrical quasiclassical method with a triangle window (SQC, triangle symbol) and the linearized semiclassical methods 1 (LSC1, circle symbol) and 2 (LSC2, square symbol). Data of the all-atom nonadiabatic SQC and LSC dynamics were obtained from ref (69).

**Figure 13:**
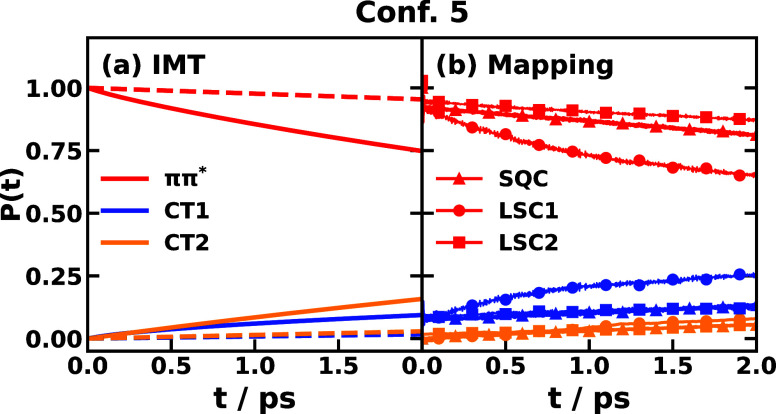
Comparison of population dynamics of CPC_60_ triad conformation 5 dissolved in explicit THF solvent at 300 K with the initial electronic state on ππ* obtained with (a) instantaneous Marcus theory (IMT, solid) and traditional Marcus theory (dashed) and (b) nonadiabatic mapping dynamics using the symmetrical quasiclassical method with a triangle window (SQC, triangle symbol) and the linearized semiclassical methods 1 (LSC1, circle symbol) and 2 (LSC2, square symbol).

Finally, we compare the population dynamics from IMT with the traditional Marcus theory as well as the all-atom nonadiabatic mapping dynamics including the symmetrical quasiclassical and the linearized semiclassical mapping dynamics for triad Confs. 3 and 5 in Figures 12 and 13, respectively. Data of the all-atom nonadiabatic SQC and LSC dynamics of Conf. 3 are obtained from ref (69), where the reader can find a detailed description of the dynamics, and the results of Conf. 5 are simulated using the same methods. Here, only starting with an electronic population on the ππ* state is considered for this comparison. For Conf. 3, it is evident that the IMT prediction agrees better with the all-atom nonadiabatic dynamics than the Marcus theory does, as shown in Figure 12, which suggests the importance of accounting for the initial nonequilibrium nuclear state. From Marcus theory, the population decay of the ππ* state is too slow compared with the nonadiabatic dynamics, which cannot give the right physical picture of the photoinduced charge transfer that happens in the 2 ps time scale. In contrast, IMT could capture the right time scale for the ππ* population decay but overestimate the population gain in the CT2 state in the long time. For Conf. 5, the comparison is shown in Figure 13. IMT is seen to agree better with the nonadiabatic mapping dynamics than the Marcus theory does, even if the nonequilibrium effects are unsubstantial for Conf. 5 starting with the ππ* population. It is also noted that the nonadiabatic mapping dynamics are believed to be more realistic in simulating the entire dynamics coherently and the nuclear dynamics would be affected by the on-the-fly reduced electronic density matrix, but they are not exact quantum-mechanical method and still approximate dynamical method. The fact that IMT could give similar predictions as the mapping dynamics and IMT requires much simpler implementation (only classical MD simulation) and less computational cost indicates the utility of IMT in studying population transfer dynamics in complex condensed-phase systems.

## 6 Concluding Remarks

In this work, we develop the multistate nonlinear-response instantaneous Marcus theory approach for studying population transfer dynamics between multiple electronic states in complex condensed-phase systems. The multistate nonlinear-response IMT takes advantage of the equilibrium molecular dynamics simulation and provides a cost-effective strategy for calculating time-dependent IMT rate coefficients of all pairwise transitions in multiple states. The underlying time correlation functions of the energy gaps of the IMT rates require canonical sampling over the equilibrated initial electronic state for each transition. The time-dependent average and variance of corresponding energy gaps could be calculated with the input of the TCFs of *C*_1_^NL^(*t*) and *C*_2_^NL^(*t*) as well as the ensemble averages and variance of corresponding energy gaps. The resulting time-dependent IMT rates for all possible transitions were used to solve the quantum master equations for obtaining population dynamics. We employed a photoinduced CT process in two prototypical OPV CPC_60_ triad conformations dissolved in explicit THF solvent as an example to test the new IMT approach. Numerical results show that multistate IMT could capture the significant nonequilibrium effects due to the initial nuclear state preparation, such as sampled from thermal equilibrium with the ground state’s PES, but the initial electronic state is the ππ* population as a result of photoexcitation. The significant nonequilibrium effects are indicated by the substantial differences between the population dynamics predicted by multistate IMT and the Marcus theory, where the Marcus theory underestimates the population transfer systematically. Moreover, the population dynamics of the triad system using multistate IMT are seen to have a better agreement with the all-atom nonadiabatic mapping dynamics than the Marcus theory does. Because multistate IMT is straightforward in implementation which only requires incoherent classical MD simulations and the nonlinear-response formulation reduces the computational cost, we believe the multistate nonlinear-response IMT will be useful in studying charge transfer dynamics in complex condensed-phase systems, such as disordered OPV materials,^6,9,10,13,14,92,93^ molecular aggregates with aggregation-induced-emission effect,^94−96^ and solar-energy-harvesting biological chromophores in complicated protein environments.^97−99^

## Supporting Information Available

Average values of ⟨*e*^*βδU*_*jg*_^⟩, schematic illustrations for energy fluctuations, time-dependent averages, variances, activation energies, CT rate coefficients, and TCFs of energy gaps as well as the molecular geometries of triad Confs. 3 and 5 (PDF)
