# Supplementary material for: All-Atom Photoinduced Charge Transfer Dynamics in Condensed Phase via Multistate Nonlinear-Response Instantaneous Marcus Theory
Source: J Chem Theory Comput. 2024 Apr 24;20(9):3993–4006. doi: 10.1021/acs.jctc.4c00010 (PMC11099976; doi:10.1021/acs.jctc.4c00010)
Supplement: Supplementary file 1 — ct4c00010_si_001.pdf [file ct4c00010_si_001.pdf]

Supporting Information: All-Atom Photoinduced Charge Transfer Dynamics in Condensed Phase via Multistate Nonlinear-Response Instantaneous Marcus Theory

Zengkui Liu,[†,‡,¶] Zailing Song,[†] and Xiang Sun[*,†,‡,¶]

†*Division of Arts and Sciences, NYU Shanghai, 567 West Yangsi Road, Shanghai 200124, China*

‡*NYU-ECNU Center for Computational Chemistry at NYU Shanghai, 3663 Zhongshan Road North, Shanghai 200062, China*

¶*Department of Chemistry, New York University, New York, New York 10003, United States*

E-mail: xiang.sun@nyu.edu

1 Supporting data for triad conformations 3 and 5

Table S1: Ensemble average of $\langle e^{-\beta U_{jg}} \rangle$ for triad conformations 3 and 5

Conf. 3	$\pi\pi^* \to$ CT1	$\pi\pi^* \to$ CT2	CT1 $\to \pi\pi^*$	CT1 $\to$ CT2	CT2 $\to \pi\pi^*$	CT2 $\to$ CT1
$\langle e^{-\beta U_{jg}} \rangle$	1.680×10^5	2.729×10^7	9.245×10^4	3.023	3.168×10^7	2.870
Conf. 5	$\pi\pi^* \to$ CT1	$\pi\pi^* \to$ CT2	CT1 $\to \pi\pi^*$	CT1 $\to$ CT2	CT2 $\to \pi\pi^*$	CT2 $\to$ CT1
$\langle e^{-\beta U_{jg}} \rangle$	1.669×10^4	9.062×10^{10}	5.076×10^4	6.413×10^{11}	2.915×10^{10}	9.520×10^{10}

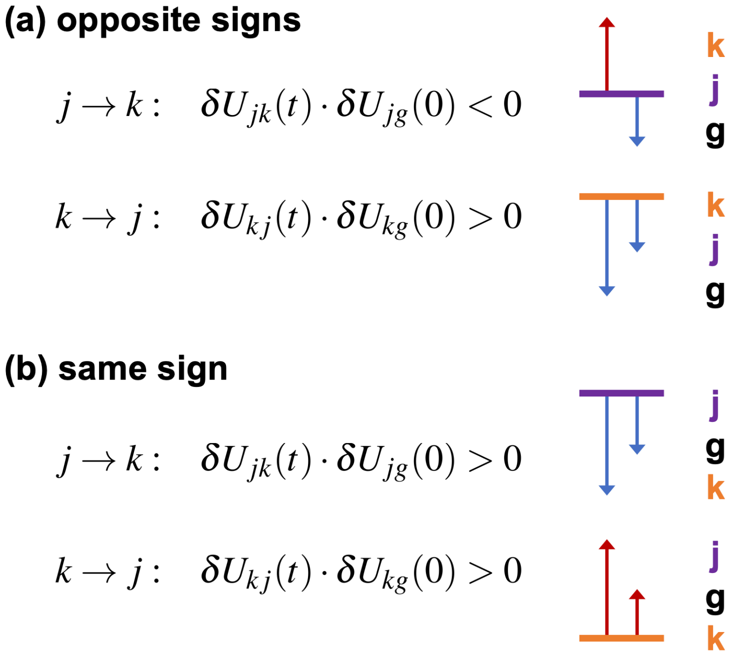

Figure S1: Schematic illustrations of the instantaneous energy fluctuations $\delta U_{jk}(t)$ and $\delta U_{jg}(0)$ that lead to (a) opposite signs and (b) same sign in $C_1^{\mathrm{NL}}(t)$ of forward ($j \to k$) and backward ($k \to j$) transitions. The absolute energy levels of j, k, g do not matter when discussing the fluctuations, so the relative heights represent the upward or downward moving speed of energy with respect to the j (purple) or k (orange) energies. The relative moving speed of moving upward and downward is indicated as a red upward arrow and a blue downward arrow, respectively.

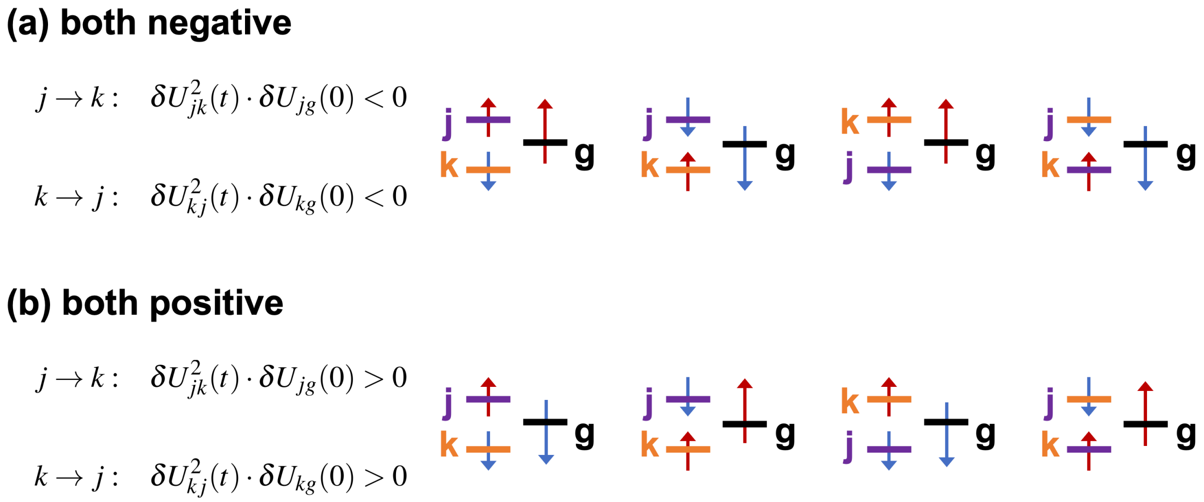

Figure S2: Schematic illustrations of the instantaneous energy fluctuations $\delta U_{jk}^2(t)$ and $\delta U_{jg}(0)$ that lead to (a) both negative sign and (b) both positive sign in $C_2^{\mathrm{NL}}(t)$ of forward ($j \to k$) and backward ($k \to j$) transitions. The absolute energy levels of j, k, g do not matter when discussing the fluctuations, so j, k separating away from each other only means instant $\delta U_{jk}^2(t) > 0$, vice versa. The relative moving speed of moving upward and downward is indicated as a red upward arrow and a blue downward arrow, respectively.

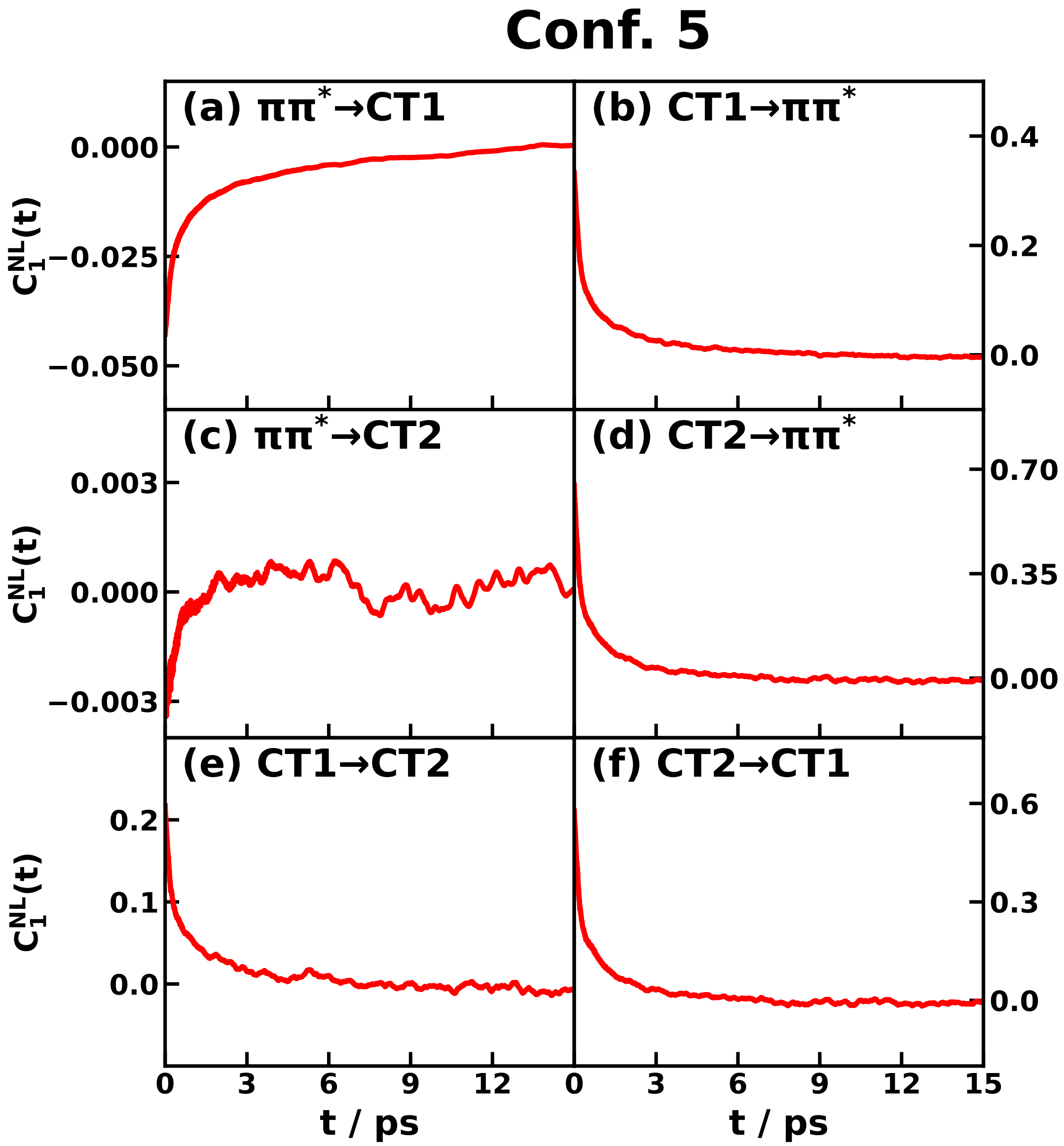

Figure S3: Time correlation function $C_1^{\mathrm{NL}}(t)$ of CPC_{60} triad conformation 5 for different transitions $j \rightarrow k$ obtained from all-atom equilibrium MD simulations on the V_j potential surface at 300 K.

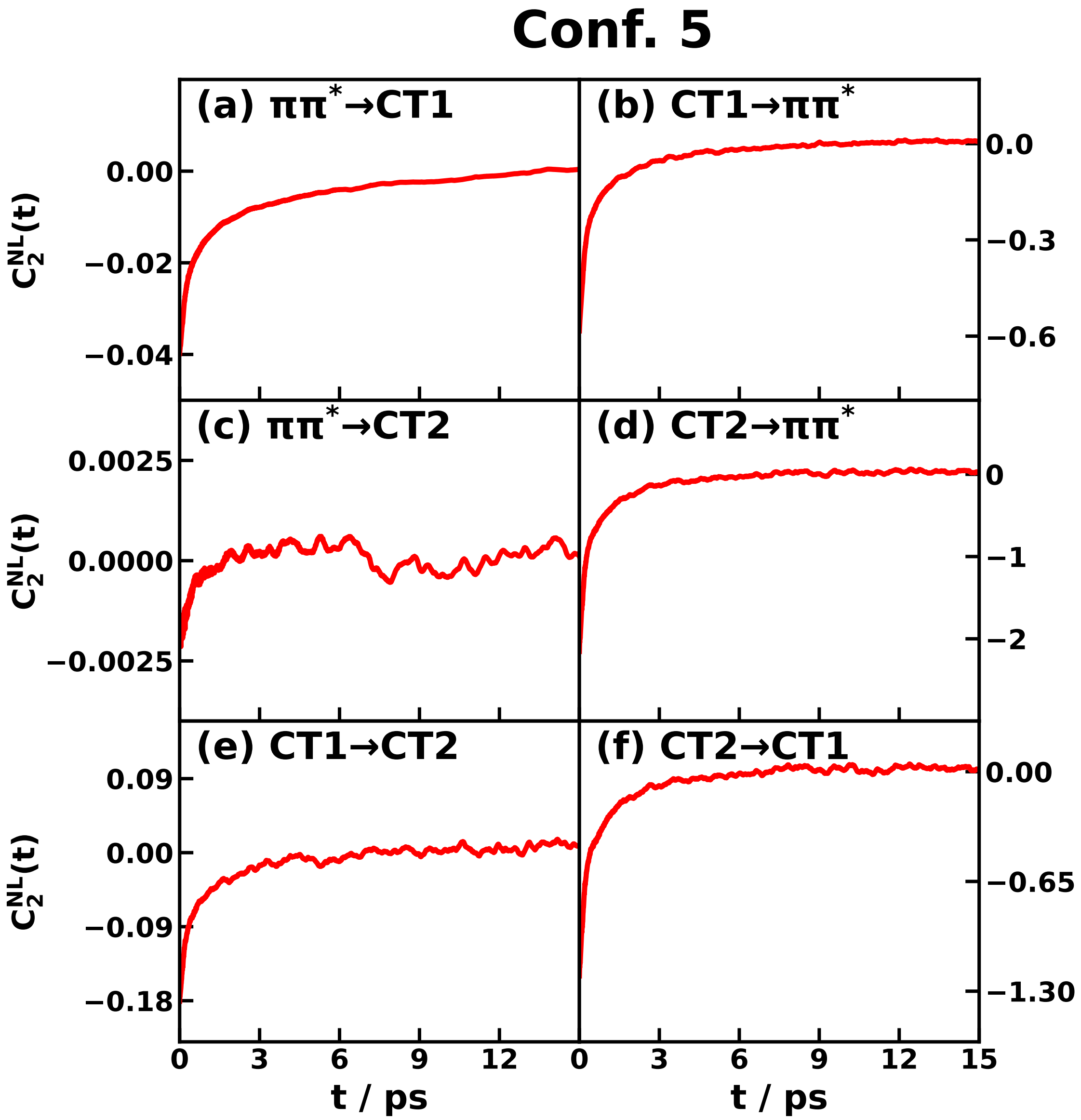

Figure S4: Time correlation function $C_2^{\mathrm{NL}}(t)$ of CPC_{60} triad conformation 5 for different transitions $j \to k$ obtained from all-atom equilibrium MD simulations on the V_j potential surface at 300 K.

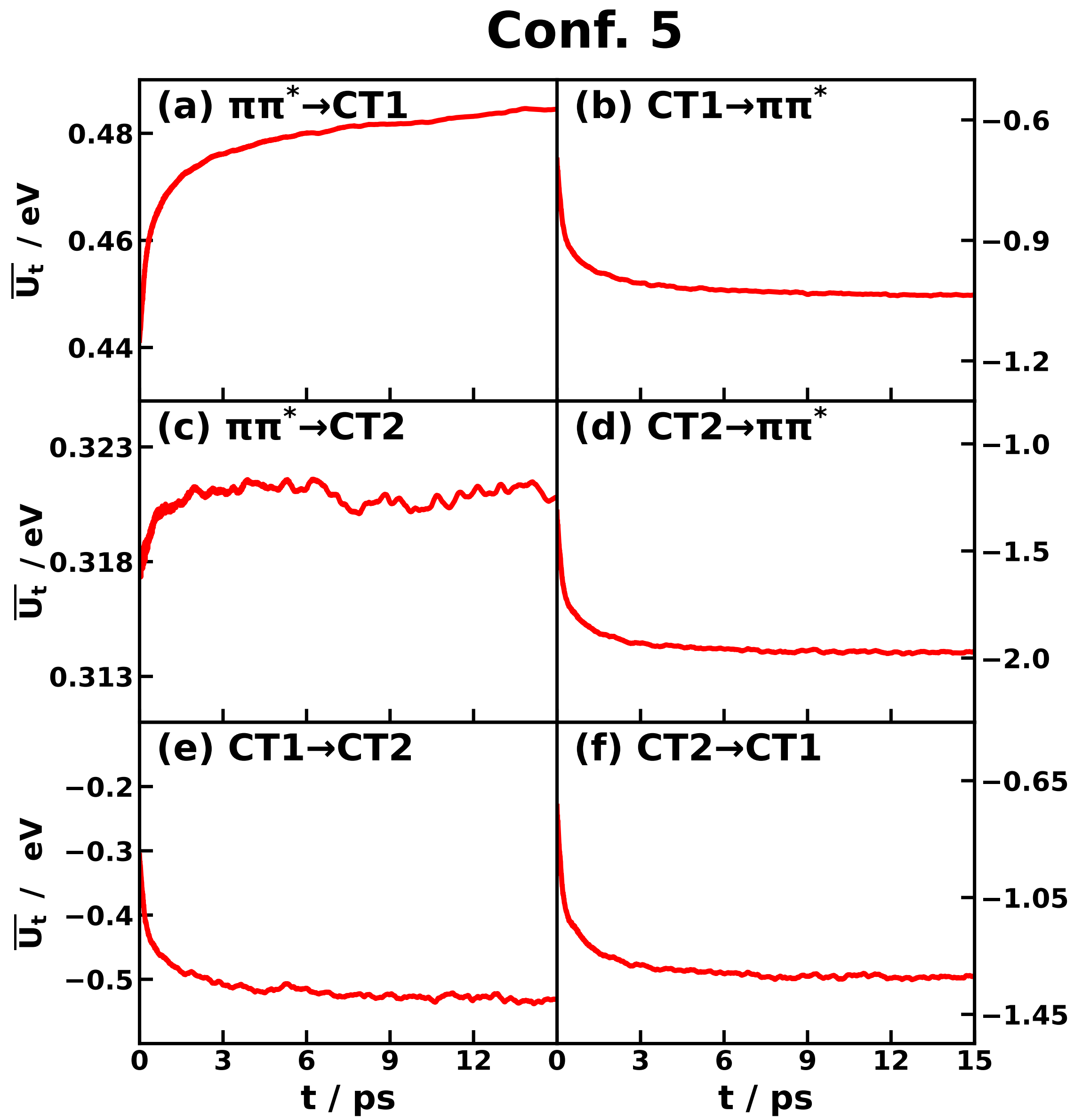

Figure S5: Time-depenent average energy gap $\overline{U(t)}$ of CPC_{60} triad conformation 5 for different transitions $j \rightarrow k$ obtained from all-atom equilibrium MD simulations on the V_j potential surface at 300 K.

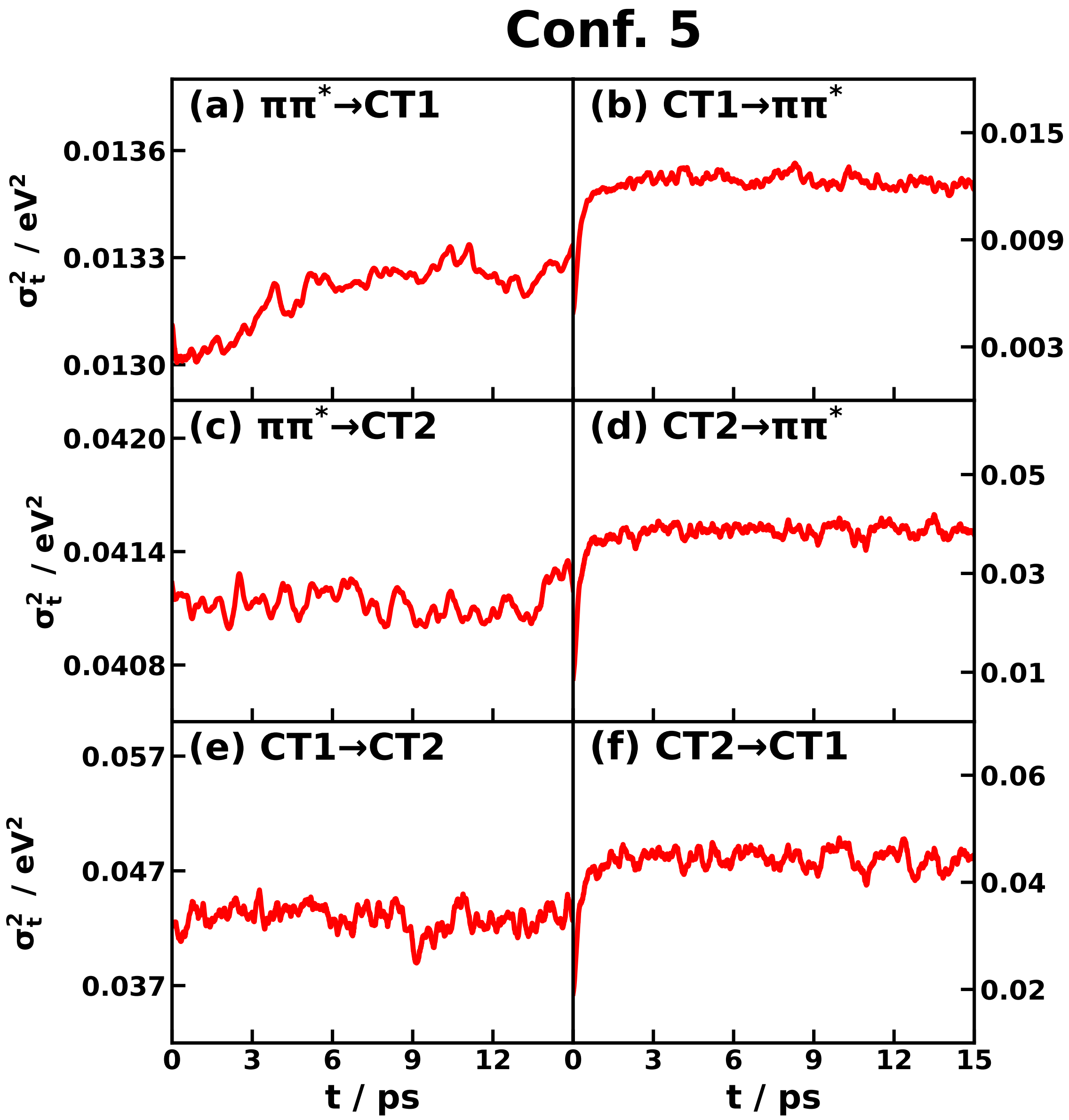

Figure S6: Time-dependent energy gap variance $\overline{\sigma^2(t)}$ of CPC_{60} triad conformation 5 for different transitions $j \rightarrow k$ obtained from all-atom equilibrium MD simulations on the V_j potential surface at 300 K.

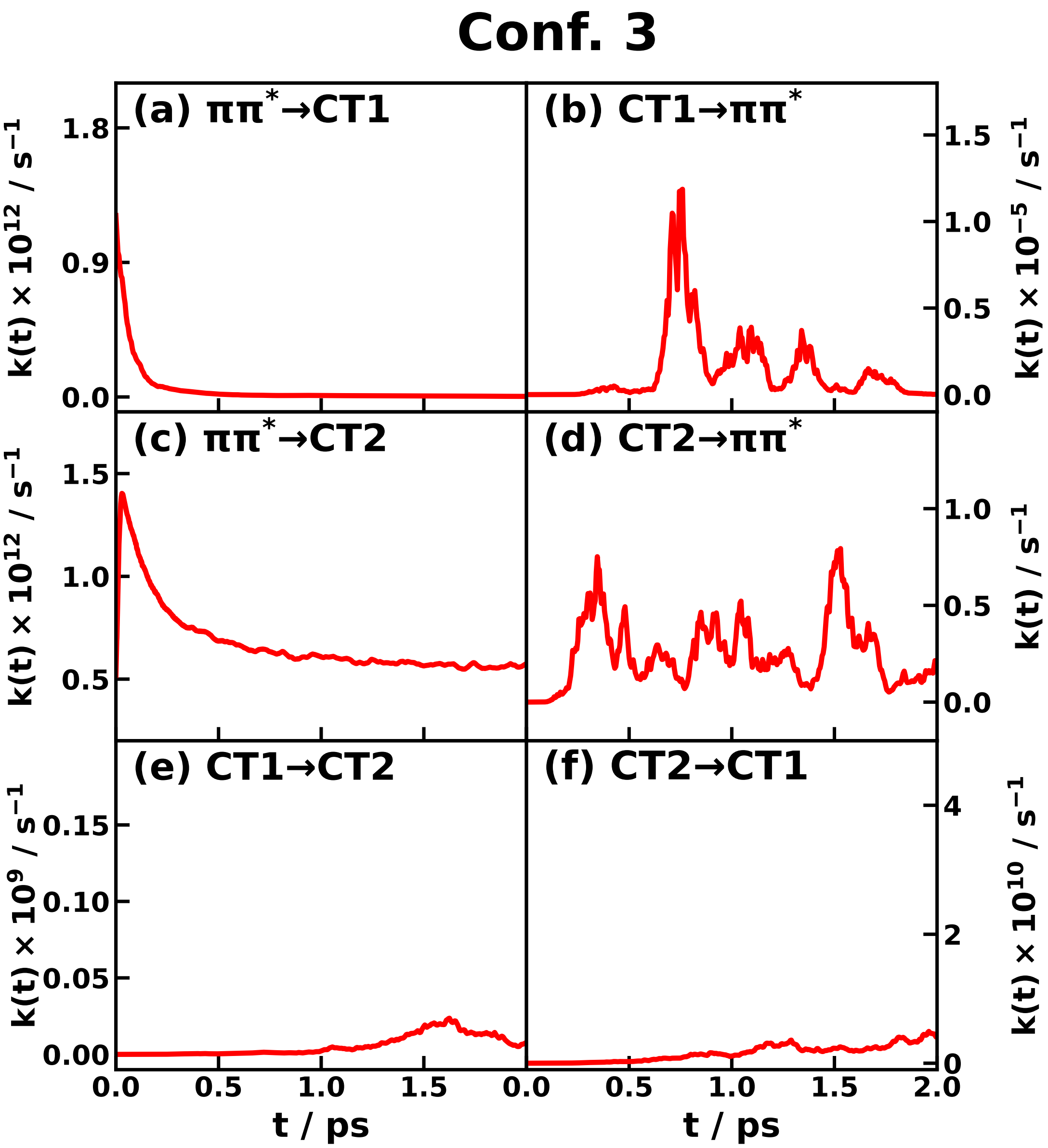

Figure S7: Short-time profile of time-dependent IMT rate coefficients of triad conformation 3 for different transitions $j \to k$ at 300 K.

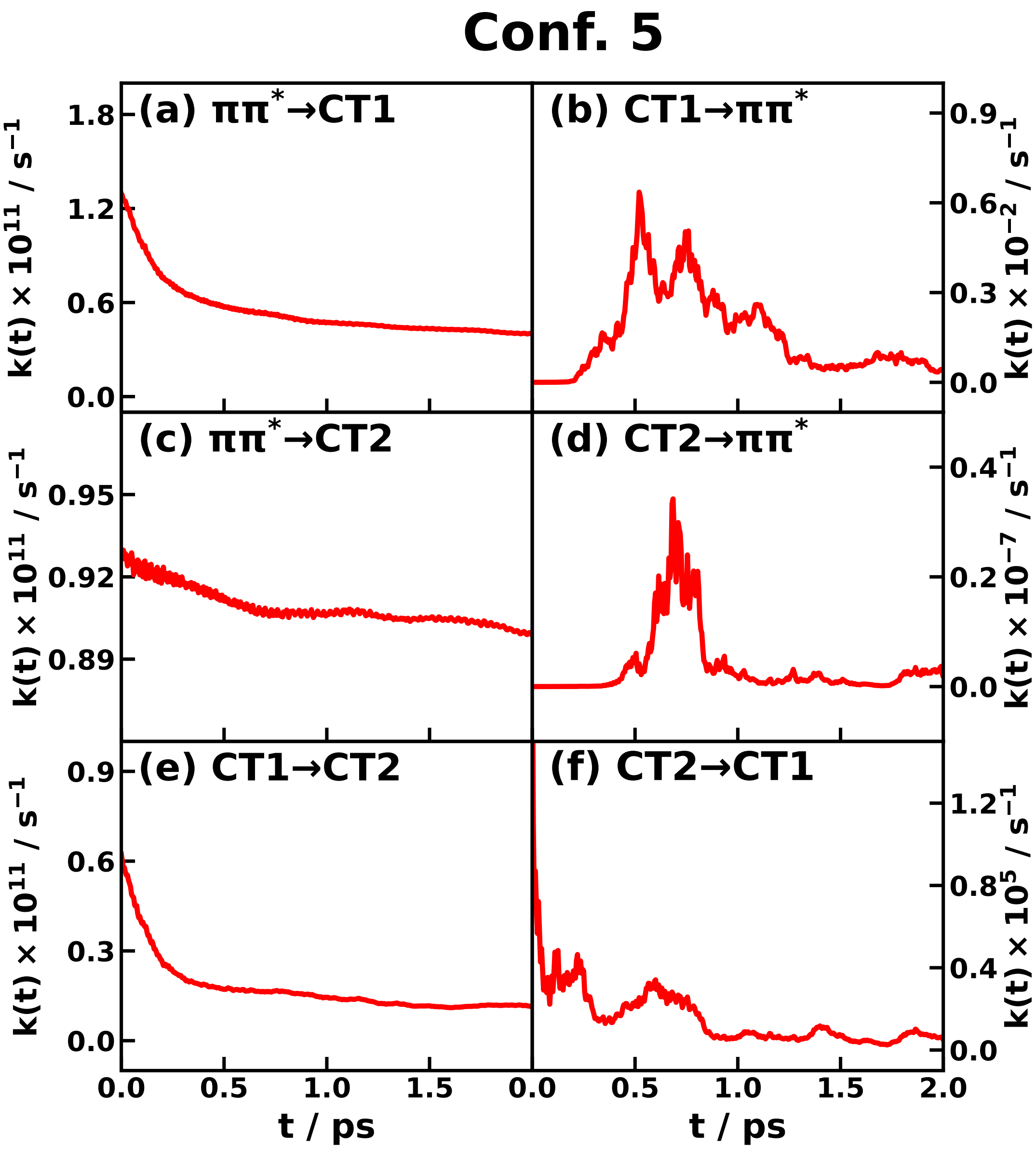

Figure S8: Short-time profile of time-dependent IMT rate coefficients of triad conformation 5 for different transitions $j \rightarrow k$ at 300 K.

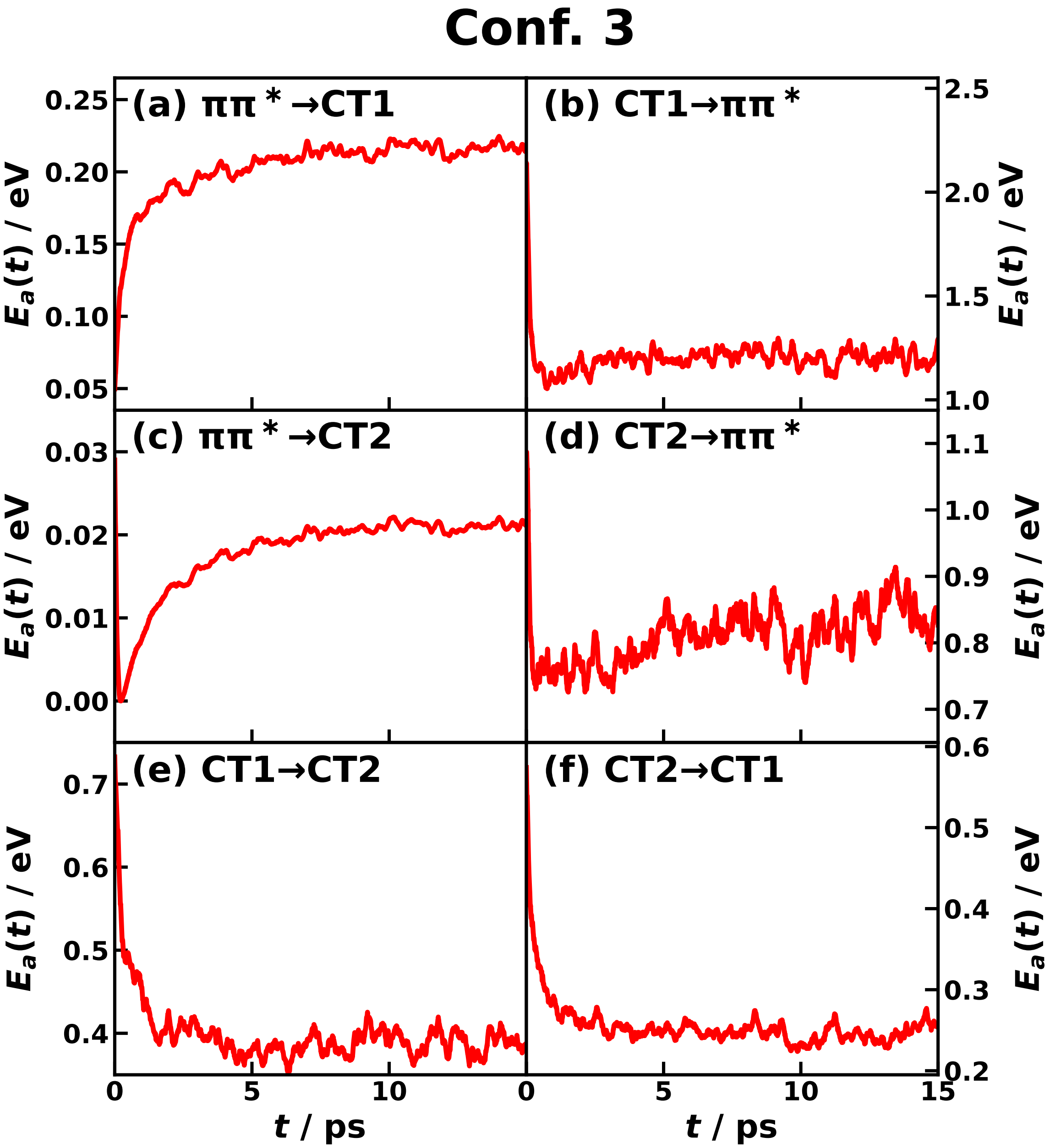

Figure S9: Time-dependent activation energy of triad conformation 3 for different transitions $j \to k$ at 300 K.

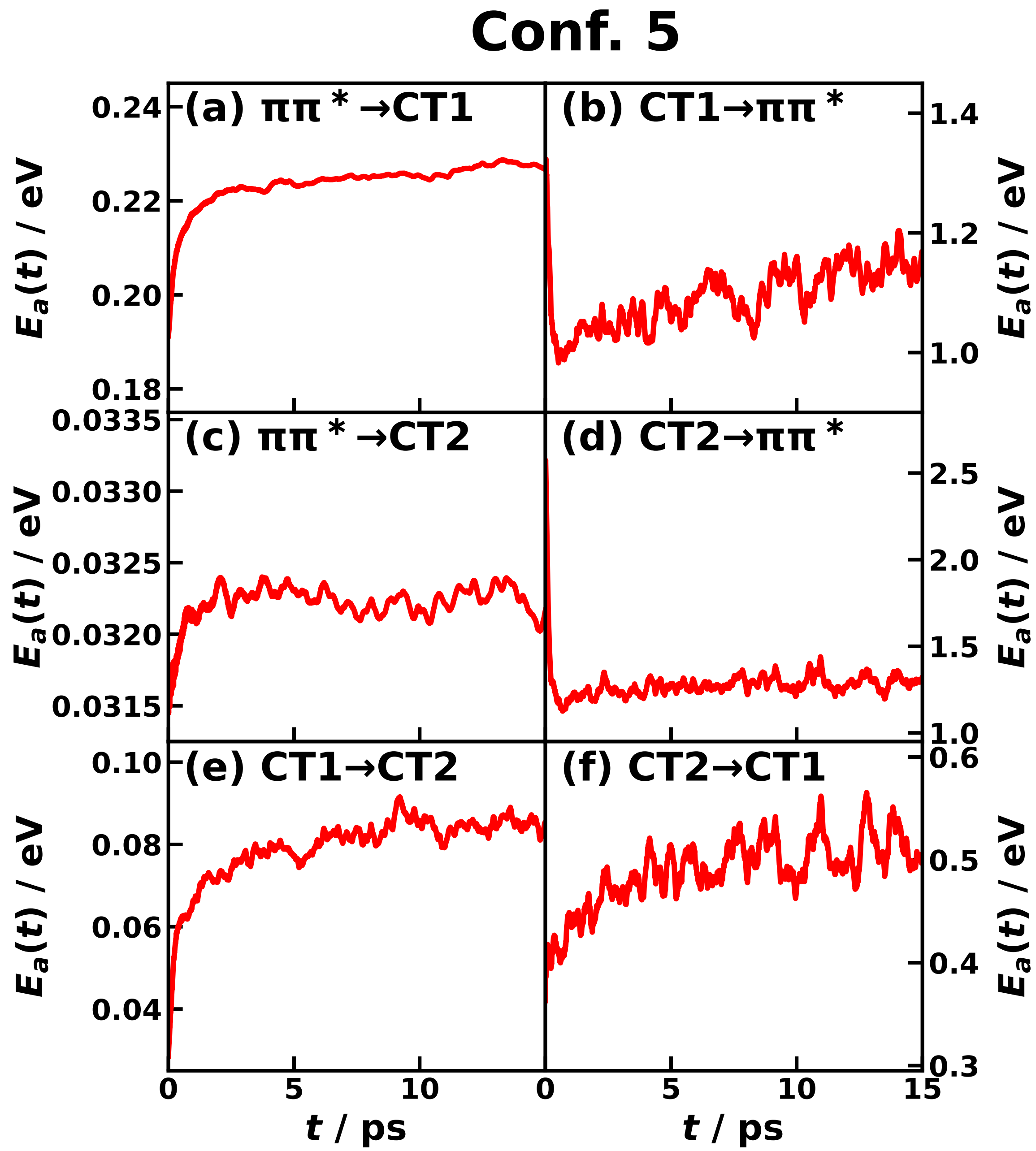

Figure S10: Time-dependent activation energy of triad conformation 5 for different transitions $j \rightarrow k$ at 300 K.

2 Supplemental figures with statistical error bars

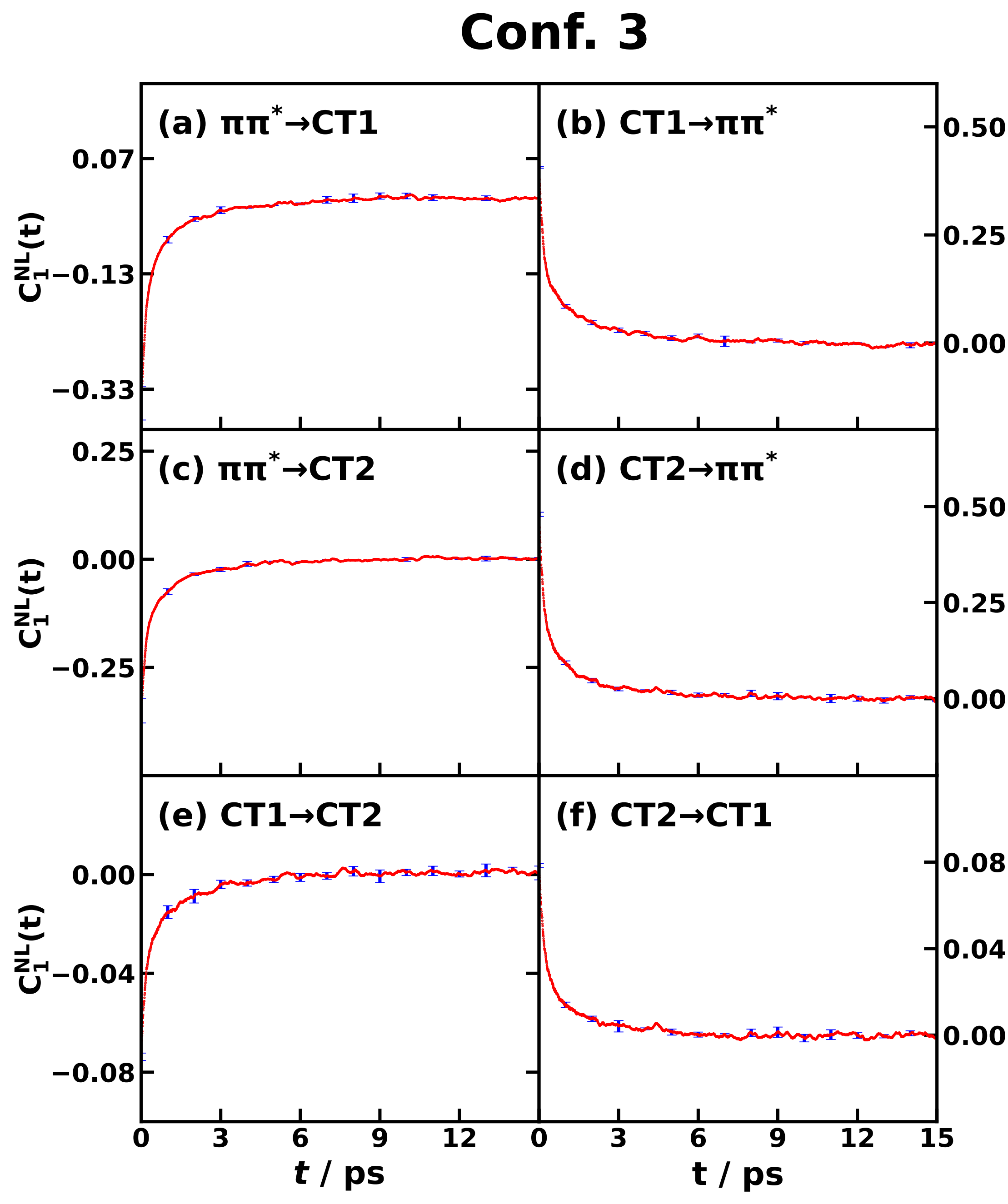

Figure S11: Time correlation function $C_1^{\mathrm{NL}}(t)$ of CPC_{60} triad conformation 3 for different transitions $j \to k$ obtained from all-atom equilibrium MD simulations on the V_j potential surface at 300 K.

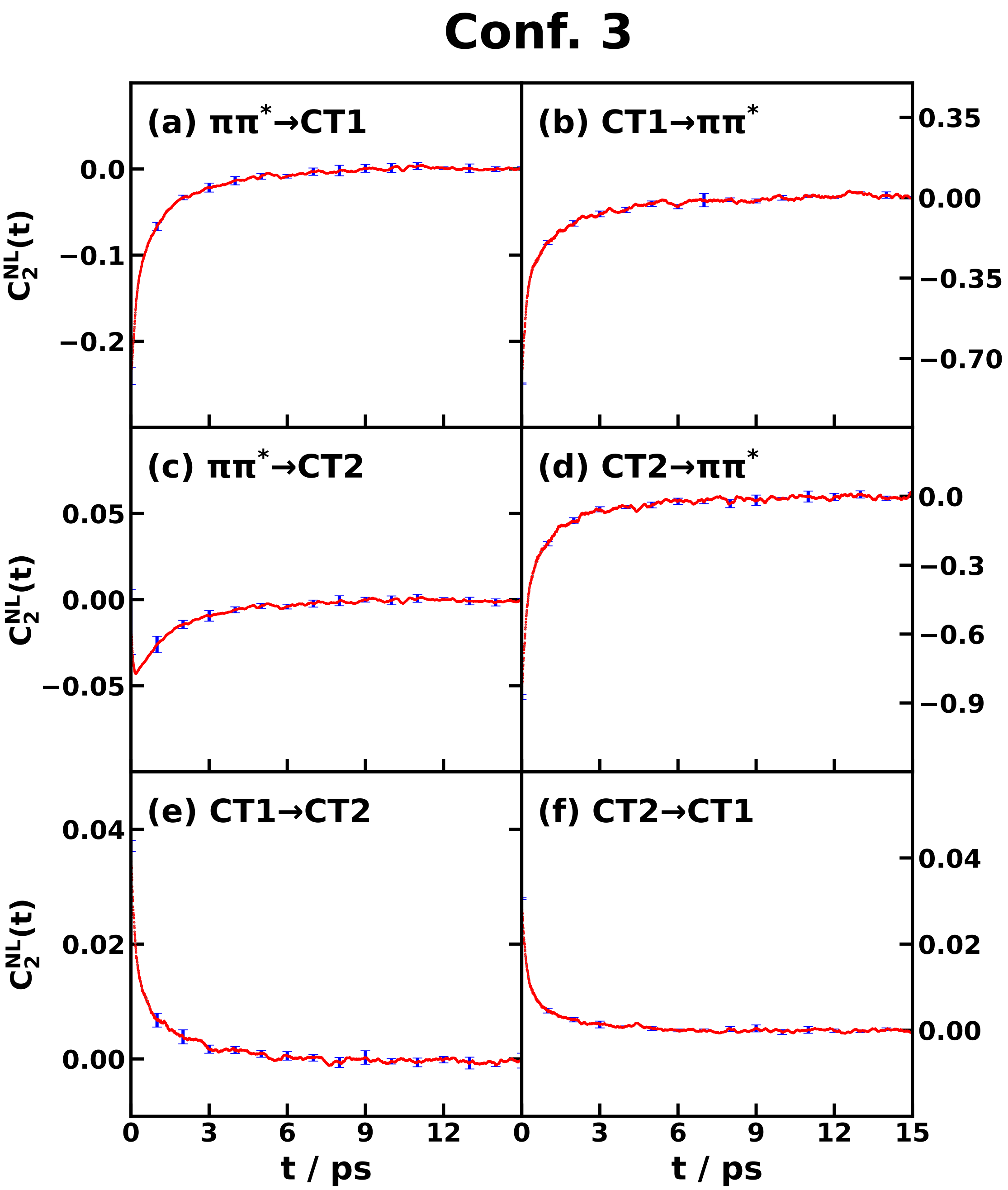

Figure S12: Time correlation function $C_2^{\mathrm{NL}}(t)$ of CPC_{60} triad conformation 3 for different transitions $j \rightarrow k$ obtained from all-atom equilibrium MD simulations on the V_j potential surface at 300 K.

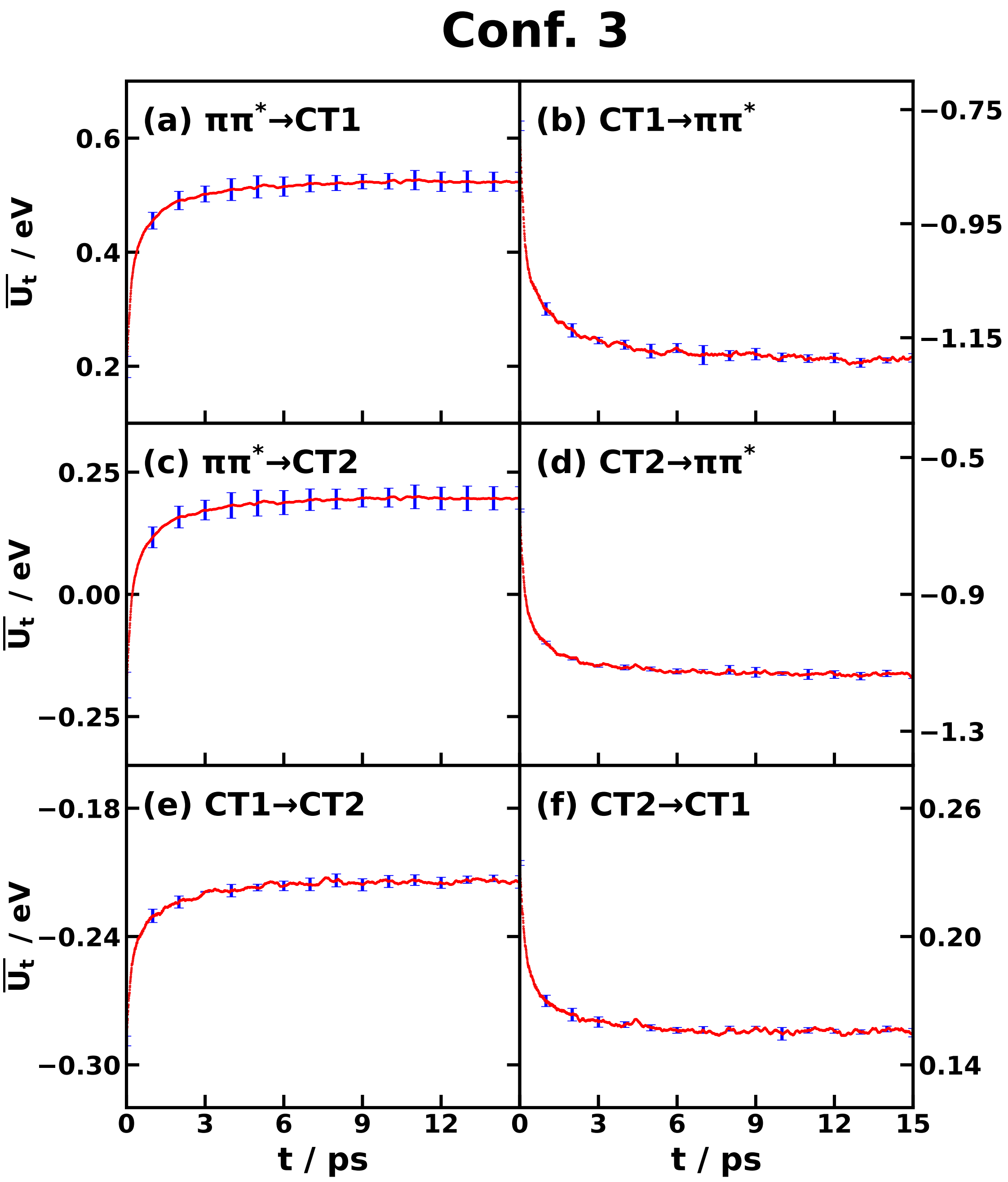

Figure S13: Time-depenent average energy gap $\overline{U(t)}$ of CPC_{60} triad conformation 3 for different transitions $j \to k$ obtained from all-atom equilibrium MD simulations on the V_j potential surface at 300 K.

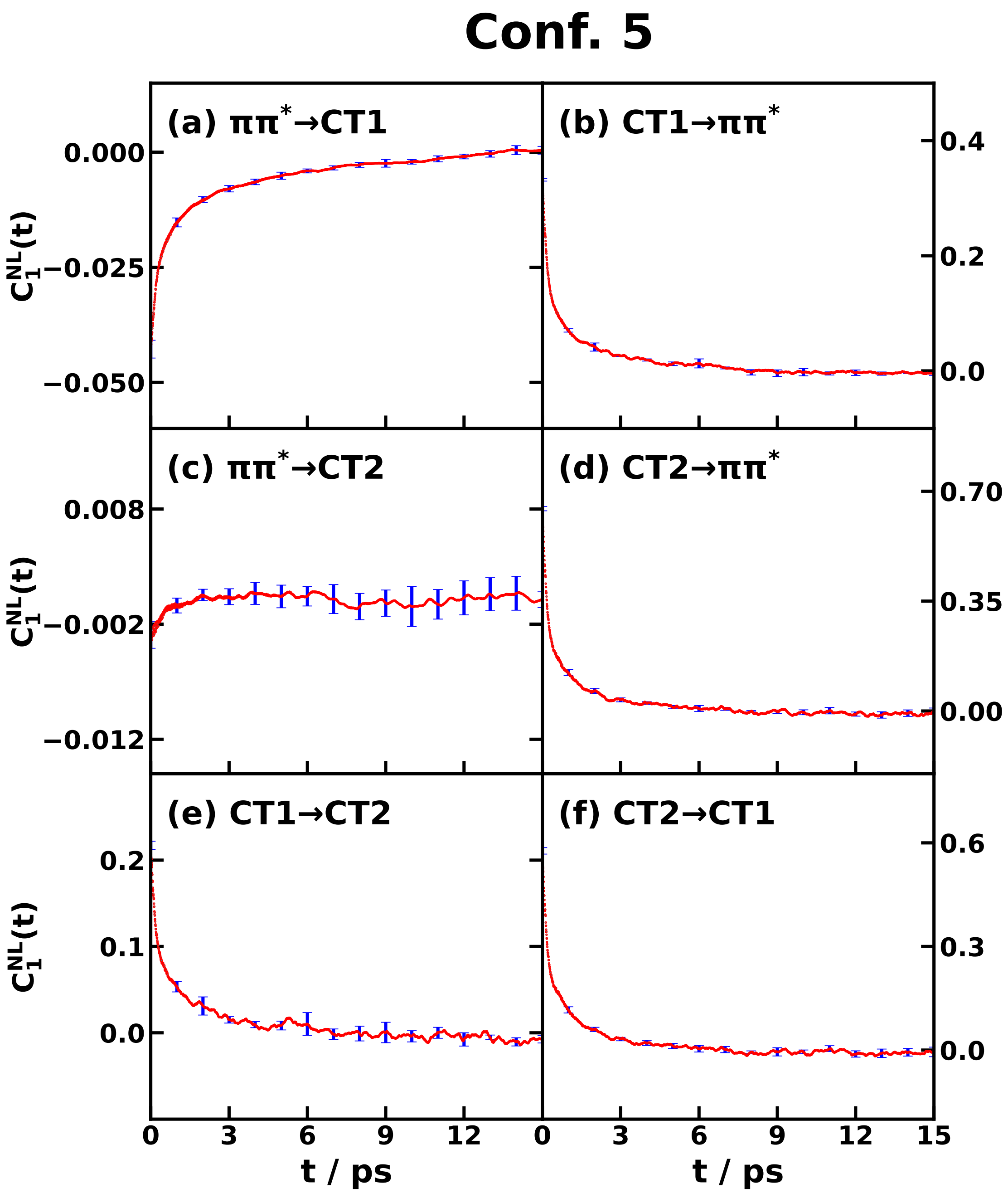

Figure S14: Time correlation function $C_1^{\mathrm{NL}}(t)$ of CPC_{60} triad conformation 5 for different transitions $j \to k$ obtained from all-atom equilibrium MD simulations on the V_j potential surface at 300 K.

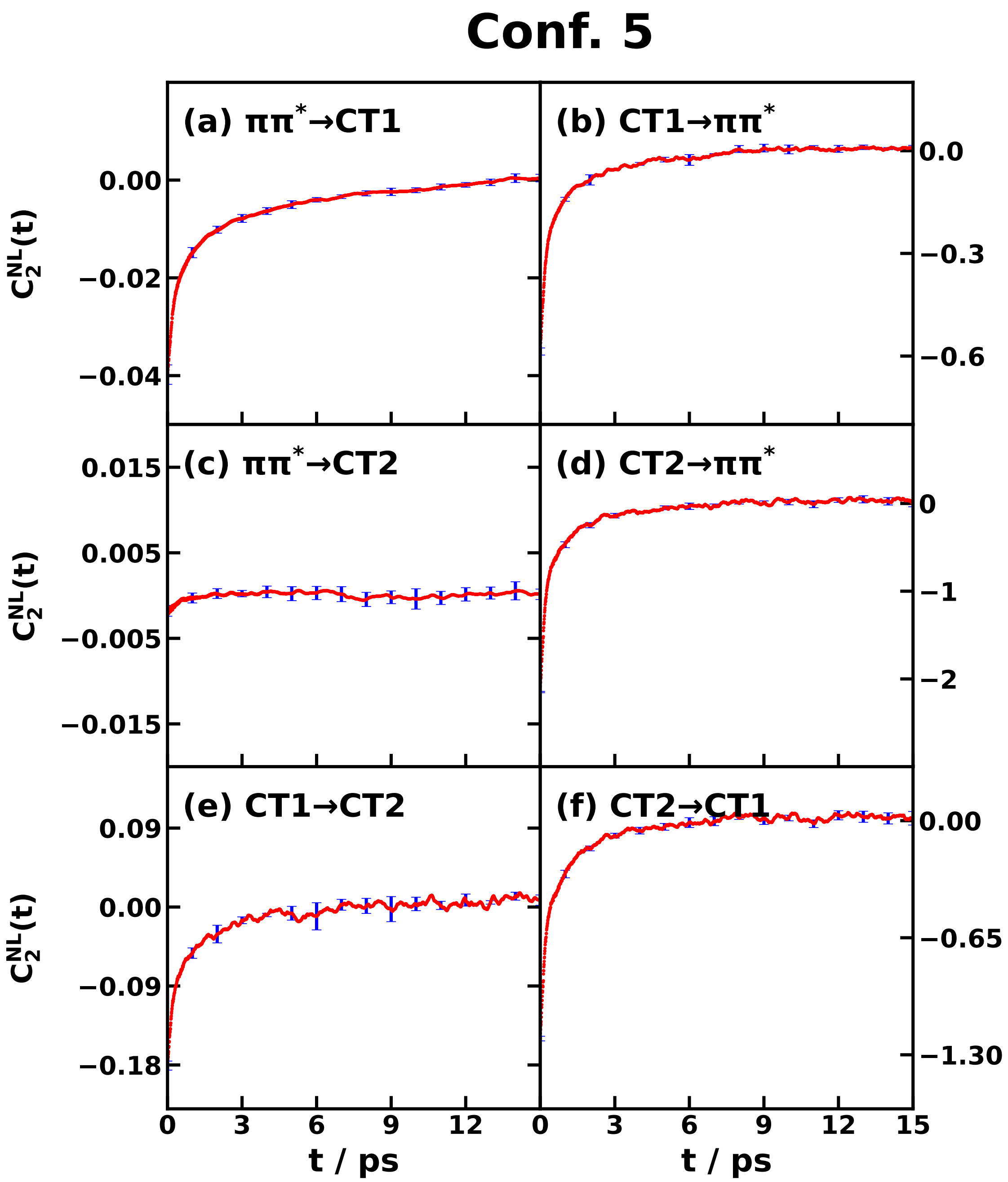

Figure S15: Time correlation function $C_2^{\mathrm{NL}}(t)$ of CPC_{60} triad conformation 5 for different transitions $j \to k$ obtained from all-atom equilibrium MD simulations on the V_j potential surface at 300 K.

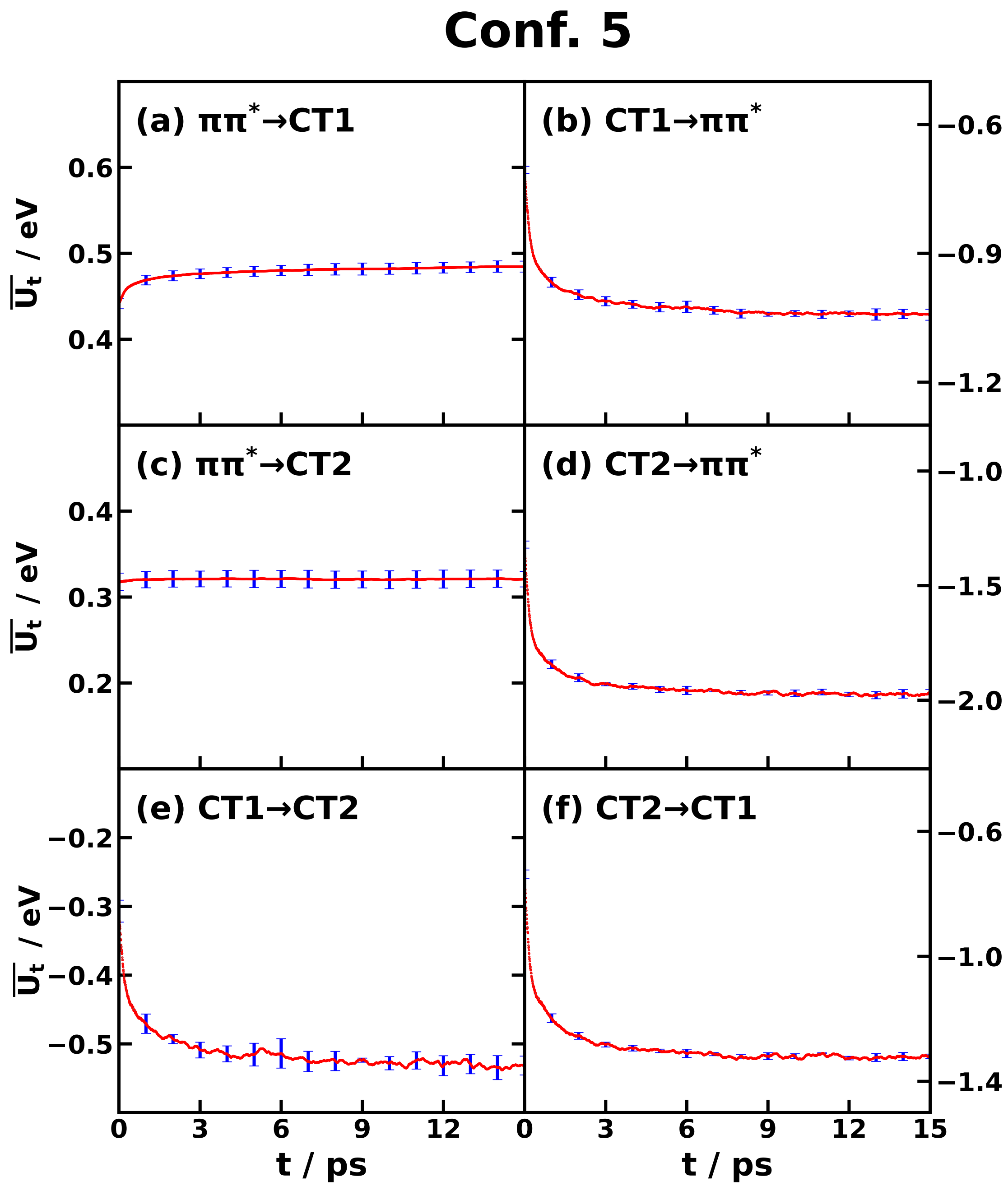

Figure S16: Time-depenent average energy gap $\overline{U(t)}$ of CPC_{60} triad conformation 5 for different transitions $j \to k$ obtained from all-atom equilibrium MD simulations on the V_j potential surface at 300 K.

3 Geometries of triad conformations 3 and 5

Table S2: Geometry of triad conformation 3

index	atom type	x (Å)	y (Å)	z (Å)
1	C	40.035423278808594	34.077476501464844	50.6230583190918
2	C	39.337501525878906	39.24565887451172	54.412235260009766
3	C	37.78224182128906	37.75056076049805	53.75490951538086
4	C	37.78740310668945	35.54536056518555	52.88954544067383
5	C	41.75084686279297	39.47233200073242	54.52494430541992
6	C	40.55373764038086	39.84507751464844	53.97770309448242
7	C	40.50603103637695	37.87956237792969	55.81369400024414
8	C	38.69609069824219	35.923255920410156	55.06357955932617
9	C	41.02073287963867	33.3150634765625	52.45847702026367
10	C	41.762672424316406	38.46281433105469	55.4941520690918
11	C	39.067054748535156	33.98578643798828	51.58659744262695
12	C	44.052730560302734	37.99654006958008	52.301151275634766
13	C	42.74968338012695	39.52995300292969	53.55015182495117
14	C	43.968379974365234	35.80602264404297	51.42007827758789
15	C	43.077571868896484	34.282615661621094	53.08613967895508
16	C	43.3669319152832	34.60298538208008	51.71572494506836
17	C	43.724578857421875	37.498966217041016	54.429080963134766
18	C	40.75470733642578	36.498355865478516	55.99897384643555
19	C	39.876216888427734	35.48761749267578	55.59810256958008
20	C	41.60490798950195	34.06745529174805	54.76492691040039
21	C	38.98292922973633	38.713382720947266	49.75773239135742
22	C	40.154541015625	39.56853103637695	50.36030960083008
23	C	38.17045211791992	38.47359085083008	51.0485954284668

24	C	38.67604446411133	39.268131256103516	52.07322311401367
25	C	42.419578552246094	35.275978088378906	49.84572219848633
26	C	41.49324417114258	39.14722442626953	50.19460678100586
27	C	38.176612854003906	35.03931427001953	51.67079544067383
28	C	43.45918655395508	38.498775482177734	51.14821243286133
29	C	42.136783599853516	39.95972442626953	52.38497543334961
30	C	43.3621940612793	36.22722625732422	50.28903579711914
31	C	37.663055419921875	37.215728759765625	51.35324478149414
32	C	44.06630325317383	36.332557678222656	53.76621627807617
33	C	43.56576156616211	35.143959045410156	54.09919357299805
34	C	40.2840576171875	34.3243522644043	55.01182174682617
35	C	42.0673828125	36.31003189086914	55.67795944213867
36	C	39.46428298950195	37.41359329223633	49.03645706176758
37	C	40.96926498413086	37.10572052001953	49.10977554321289
38	C	39.02239227294922	36.045467376708984	49.66830062866211
39	C	41.188148498535156	35.70775604248047	49.28049087524414
40	C	41.95845031738281	33.59429168701172	53.46261215209961
41	C	39.78782272338867	39.95668029785156	51.67953872680664
42	C	38.16611862182617	36.07628631591797	50.7779426574707
43	C	39.980743408203125	35.1283073425293	49.67337417602539
44	C	37.477596282958984	36.88923263549805	52.71952438354492
45	C	42.43134307861328	34.23780059814453	50.772281646728516
46	C	41.887611389160156	38.02587890625	49.57029342651367
47	C	42.49321365356445	39.42333221435547	51.20346450805664
48	C	38.455326080322266	38.92343521118164	53.41828536987305
49	C	40.765724182128906	40.190425872802734	52.63469314575195
50	C	42.539161682128906	35.102970123291016	55.1218376159668

51	C	43.07819747924805	37.62501525878906	50.162960052490234
52	C	41.22753143310547	33.642478942871094	51.14430618286133
53	C	39.31019592285156	38.19858932495117	55.26387405395508
54	C	38.403709411621094	37.25876235961914	54.884281158447266
55	C	38.32035827636719	35.05776596069336	54.058528900146484
56	C	39.66483688354492	33.52842330932617	52.76191329956055
57	C	43.74524688720703	38.54375457763672	53.53744888305664
58	C	44.258888244628906	36.65840148925781	52.452816009521484
59	C	39.3271598815918	34.02342987060547	54.02800369262695
60	C	42.735164642333984	37.463417053222656	55.40768814086914
61	C	38.0670280456543	39.350379943847656	48.68034744262695
62	N	38.04104232788086	38.69755172729492	47.567508697509766
63	C	38.893699645996094	37.50092697143555	47.58279800415039
64	H	39.76797866821289	37.57107162475586	46.93059158325195
65	H	38.25526428222656	36.62056350708008	47.473140716552734
66	C	36.89878845214844	43.54693603515625	48.445377349853516
67	C	37.06329345703125	42.91847229003906	49.71615982055664
68	H	36.87632751464844	43.387237548828125	50.678897857666016
69	C	37.452030181884766	41.558998107910156	49.70392990112305
70	H	37.35005187988281	41.00393295288086	50.6329460144043
71	C	37.686092376708984	40.8308219909668	48.57065200805664
72	C	37.169437408447266	42.84180450439453	47.25648880004883
73	H	37.23537063598633	43.31881332397461	46.2819709777832
74	C	37.573524475097656	41.46322250366211	47.305728912353516
75	H	37.87754440307617	40.828163146972656	46.47757339477539
76	C	36.44705581665039	44.98897933959961	48.45491409301758
77	C	36.16180419921875	45.673439025878906	49.708560943603516

78	C	35.2735481262207	45.41484069824219	50.753719329833984
79	H	34.572322845458984	44.59514236450195	50.87034225463867
80	C	35.41279602050781	46.466617584228516	51.70744705200195
81	H	34.77893829345703	46.654258728027344	52.56782150268555
82	C	36.50772476196289	47.21390151977539	51.267337799072266
83	N	36.878456115722656	46.728694915771484	50.070064544677734
84	H	37.674137115478516	47.1329231262207	49.59507369995117
85	C	36.98184585571289	48.44858169555664	51.783321380615234
86	H	36.54607391357422	48.7651481628418	52.729793548583984
87	C	37.95463943481445	49.27117919921875	51.28208541870117
88	C	38.355430603027344	50.44987869262695	51.945011138916016
89	H	38.01856994628906	50.79148864746094	52.91817092895508
90	C	39.18561935424805	51.052940368652344	51.0890998840332
91	H	39.803245544433594	51.90899658203125	51.339969635009766
92	C	39.37937545776367	50.12907028198242	49.93753433227539
93	N	38.48263168334961	49.13467788696289	50.026004791259766
94	C	40.178688049316406	50.3927001953125	48.7626838684082
95	C	39.64580154418945	50.27010726928711	47.48015213012695
96	C	39.6684455871582	51.11004638671875	46.3260612487793
97	H	40.2832145690918	51.976497650146484	46.10573959350586
98	C	38.72100067138672	50.62192916870117	45.4483757019043
99	H	38.51445007324219	51.08549880981445	44.489376068115234
100	C	38.16280746459961	49.40251541137695	45.891483306884766
101	N	38.691280364990234	49.317691802978516	47.1224250793457
102	H	38.50345230102539	48.4906120300293	47.67267990112305
103	C	37.38438034057617	48.542015075683594	45.2974739074707
104	H	37.17817306518555	48.78105163574219	44.2552375793457

105	C	36.8882942199707	47.28388977050781	45.80862808227539
106	C	35.94205856323242	46.36751174926758	45.280391693115234
107	H	35.46015548706055	46.60828399658203	44.33857345581055
108	C	35.6696891784668	45.372188568115234	46.18452072143555
109	H	34.871585845947266	44.645511627197266	46.07404708862305
110	C	36.52386474609375	45.62120819091797	47.21135330200195
111	N	37.224945068359375	46.71147918701172	46.99198532104492
112	C	44.369869232177734	51.21619415283203	49.516361236572266
113	C	43.90370178222656	51.25489807128906	48.21174240112305
114	H	44.59870147705078	51.55831527709961	47.43296432495117
115	C	42.53765106201172	50.96190643310547	48.01423263549805
116	H	42.113033294677734	50.932647705078125	47.0140266418457
117	C	41.62492752075195	50.589962005615234	49.05727005004883
118	C	43.56096649169922	50.821311950683594	50.56925582885742
119	H	44.05266189575195	50.839473724365234	51.53852462768555
120	C	42.20302200317383	50.496116638183594	50.382503509521484
121	H	41.69416809082031	50.16132354736328	51.28281021118164
122	N	45.71440505981445	51.577213287353516	49.82648849487305
123	H	46.244136810302734	50.8552131652832	50.291439056396484
124	C	46.36732482910156	52.72185134887695	49.43257522583008
125	O	45.72726821899414	53.50487518310547	48.71463394165039
126	C	47.766815185546875	52.9835205078125	49.91049575805664
127	C	48.633323669433594	53.67185592651367	49.12913131713867
128	H	48.19963836669922	53.94063949584961	48.169315338134766
129	C	49.93336486816406	53.922210693359375	49.47035598754883
130	C	50.28663635253906	53.6131591796875	50.82182693481445
131	H	51.28923034667969	53.897029876708984	51.13132858276367

132	C	49.519805908203125	52.8598289489746	51.641292572021484
133	H	49.819252014160156	52.622100830078125	52.65883255004883
134	C	48.22178649902344	52.60742950439453	51.239986419677734
135	H	47.58567810058594	51.941429138183594	51.817378997802734
136	C	50.909019470214844	54.494930267333984	48.55067825317383
137	H	50.39117431640625	54.64427185058594	47.60438919067383
138	C	52.22270202636719	54.807456970214844	48.731693267822266
139	H	52.595680236816406	54.616661071777344	49.736881256103516
140	C	53.211830139160156	55.249267578125	47.76457595825195
141	C	52.74805450439453	55.122764587402344	46.3477668762207
142	H	51.90812683105469	54.43427276611328	46.23390579223633
143	H	53.480873107910156	54.5973014831543	45.731868743896484
144	H	52.61479187011719	56.14244079589844	45.98039627075195
145	C	54.51605224609375	55.465789794921875	48.02102279663086
146	H	54.784942626953125	55.477638244628906	49.076236724853516
147	C	55.51875305175781	55.71233367919922	47.004390716552734
148	H	55.24200439453125	55.842308044433594	45.95919418334961
149	C	56.78703308105469	56.00322723388672	47.27045822143555
150	H	57.0867919921875	55.93419647216797	48.31511306762695
151	C	57.9219970703125	56.396202087402344	46.52116012573242
152	C	58.24413299560547	55.59984588623047	45.27607345581055
153	H	57.48162078857422	54.82105255126953	45.208980560302734
154	H	58.25471496582031	56.28501892089844	44.425838470458984
155	H	59.156280517578125	55.02613067626953	45.4530143737793
156	C	58.82433319091797	57.288818359375	47.00053787231445
157	H	58.454505920410156	57.894142150878906	47.82682418823242
158	C	60.03949737548828	57.713287353515625	46.273189544677734

159	H	60.19554138183594	57.487213134765625	45.21940994262695
160	C	60.98150634765625	58.49263000488281	46.90291213989258
161	H	60.864830017089844	58.78790283203125	47.94460678100586
162	C	62.326637268066406	58.96875	46.39689254760742
163	H	62.437828063964844	58.71282196044922	45.3442497253418
164	C	63.213714599609375	59.808860778808594	46.955013275146484
165	C	62.86375427246094	60.51073455810547	48.28982925415039
166	H	61.871131896972656	60.956336975097656	48.1971321105957
167	H	62.649986267089844	59.84564208984375	49.12913131713867
168	H	63.633460998535156	61.15901184082031	48.71379470825195
169	C	64.38842010498047	60.27434539794922	46.20400619506836
170	H	64.50337219238281	59.91643524169922	45.181941986083984
171	C	65.55711364746094	60.80388641357422	46.6505241394043
172	H	65.69017028808594	60.795509338378906	47.731327056884766
173	C	66.80420684814453	61.04448699951172	45.847103118896484
174	H	66.7086410522461	60.85149383544922	44.779605865478516
175	C	68.01206970214844	61.446449279785156	46.290462493896484
176	C	68.46637725830078	61.78742218017578	47.63762283325195
177	H	67.59011840820312	61.591026306152344	48.258968353271484
178	H	69.20626068115234	60.99104309082031	47.74164962768555
179	H	68.8741226196289	62.799766540527344	47.67439651489258
180	C	69.10844421386719	61.690155029296875	45.320865631103516
181	H	69.971435546875	62.242584228515625	45.689640045166016
182	C	69.15345764160156	61.22742462158203	44.06552505493164
183	H	68.35615539550781	60.60469055175781	43.66250228881836
184	C	70.29295349121094	61.534942626953125	43.14235305786133
185	C	71.58451843261719	61.39344024658203	43.386783599853516

186	C	72.1784896850586	60.783355712890625	44.687076568603516
187	H	72.9077377319336	59.981163024902344	44.55612564086914
188	H	72.75205993652344	61.50016784667969	45.27840042114258
189	H	71.40128326416016	60.303550720214844	45.28557205200195
190	C	72.6597671508789	61.73040008544922	42.44926071166992
191	H	73.50138854980469	61.03507995605469	42.423526763916016
192	H	72.93914031982422	62.69541931152344	42.87723159790039
193	C	72.20869445800781	61.83429718017578	41.02488327026367
194	H	72.14167022705078	60.89094543457031	40.47893142700195
195	H	72.96687316894531	62.29833984375	40.3906135559082
196	C	70.93247985839844	62.65050506591797	40.928768157958984
197	H	71.0923843383789	63.659698486328125	41.31404495239258
198	H	70.63317108154297	62.774085998535156	39.88589096069336
199	C	69.8223876953125	61.97444152832031	41.70143508911133
200	C	68.68982696533203	62.93394470214844	41.81045150756836
201	H	68.92113494873047	63.86015319824219	42.340633392333984
202	H	68.38965606689453	63.23284912109375	40.803958892822266
203	H	67.76383209228516	62.57640075683594	42.26560592651367
204	C	69.29171752929688	60.796409606933594	40.826053619384766
205	H	69.03987884521484	61.12541198730469	39.81571578979492
206	H	70.06657409667969	60.03034973144531	40.898258209228516
207	H	68.39423370361328	60.34364318847656	41.25264358520508

Table S3: Geometry of triad conformation 5

index	atom type	x (Å)	y (Å)	z (Å)
1	C	47.069637298583984	52.5166015625	52.42736053466797

2	C	45.703731536865234	46.678958892822266	50.179290771484375
3	C	44.426429748535156	48.310943603515625	50.998680114746094
4	C	44.24566650390625	50.659576416015625	51.36589050292969
5	C	47.96582794189453	46.38277053833008	49.28561782836914
6	C	47.012962341308594	46.192134857177734	50.2554931640625
7	C	46.308170318603516	47.74235153198242	48.130088806152344
8	C	44.584800720214844	49.725563049316406	49.12356948852539
9	C	47.4013557434082	52.892799377441406	50.24932098388672
10	C	47.63475036621094	47.25330352783203	48.246036529541016
11	C	45.80717849731445	52.432403564453125	51.785797119140625
12	C	50.827999114990234	48.36329650878906	50.41994857788086
13	C	49.22036361694336	46.56140899658203	49.91139221191406
14	C	50.81344223022461	50.862213134765625	50.79580307006836
15	C	49.33304214477539	51.88044357299805	49.25974655151367
16	C	49.97931671142578	51.932411193847656	50.48420333862305
17	C	49.7777214050293	48.425559997558594	48.40688705444336
18	C	46.41250228881836	49.043949127197266	47.685665130615234
19	C	45.511985778808594	50.040889739990234	48.17564392089844
20	C	47.35371780395508	51.55499267578125	48.25592041015625
21	C	46.75276565551758	48.27493667602539	54.3785514831543
22	C	47.726783752441406	47.36980056762695	53.62053298950195
23	C	45.59138488769531	48.18523406982422	53.51187515258789
24	C	45.727508544921875	47.18467330932617	52.60045623779297
25	C	49.70242691040039	51.54783248901367	52.63549041748047
26	C	49.09062957763672	47.69187545776367	53.37917709350586
27	C	44.96660232543945	51.39340591430664	52.25888442993164
28	C	50.53999328613281	48.15696334838867	51.75236129760742

29	C	48.97516632080078	46.412742614746094	51.30669403076172
30	C	50.6298713684082	50.58771896362305	52.14516830444336
31	C	44.83586883544922	49.33020782470703	53.091461181640625
32	C	50.21098709106445	49.723209381103516	48.705875396728516
33	C	49.4240608215332	50.8283805847168	48.40131378173828
34	C	46.034400939941406	51.25808334350586	48.47819519042969
35	C	47.757911682128906	49.33435821533203	47.36872482299805
36	C	47.29697036743164	49.72214126586914	54.698333740234375
37	C	48.73769760131836	49.977256774902344	54.181968688964844
38	C	46.52935028076172	50.84150314331055	54.01484298706055
39	C	48.76230239868164	51.25519943237305	53.64028549194336
40	C	48.05315399169922	52.35398483276367	49.12742614746094
41	C	46.99983215332031	46.705955505371094	52.678138732910156
42	C	45.366695404052734	50.57431411743164	53.33286666870117
43	C	47.430274963378906	51.75008010864258	53.5167121887207
44	C	44.21334457397461	49.354305267333984	51.885772705078125
45	C	49.31987380981445	52.338226318359375	51.62674331665039
46	C	49.533477783203125	48.9690055847168	53.61628341674805
47	C	49.68000793457031	47.203643798828125	52.22610855102539
48	C	45.12968826293945	47.20644760131836	51.344337463378906
49	C	47.610755920410156	46.15376663208008	51.53279113769531
50	C	48.19469451904297	50.62525939941406	47.7514762878418
51	C	50.50538635253906	49.28962707519531	52.62665557861328
52	C	48.00750732421875	52.83586120605469	51.51080322265625
53	C	45.38080978393555	47.47744369506836	49.10806655883789
54	C	44.55260467529297	48.47731018066406	49.645782470703125
55	C	44.51604080200195	50.835140228271484	50.003761291503906

56	C	46.018470764160156	52.635780334472656	50.44807815551758
57	C	50.156463623046875	47.55757141113281	49.46015167236328
58	C	50.88131332397461	49.709651947021484	49.95359420776367
59	C	45.352020263671875	51.847930908203125	49.51586151123047
60	C	48.52648162841797	48.268394470214844	47.81624984741211
61	C	46.55290222167969	47.66389846801758	55.77652359008789
62	N	46.86338424682617	48.48561096191406	56.73060607910156
63	C	47.10466766357422	49.843345642089844	56.21763229370117
64	H	47.988094329833984	50.27644348144531	56.69369125366211
65	H	46.302734375	50.451847076416016	56.64339828491211
66	C	46.75177001953125	43.410621643066406	56.15110397338867
67	C	45.62063980102539	44.013221740722656	55.58863830566406
68	H	44.87841796875	43.438079833984375	55.041019439697266
69	C	45.53600311279297	45.44397735595703	55.417823791503906
70	H	44.69019317626953	46.01007080078125	55.036102294921875
71	C	46.51445007324219	46.23347473144531	55.97285842895508
72	C	47.69477462768555	44.262901306152344	56.796417236328125
73	H	48.631649017333984	43.83138656616211	57.13939666748047
74	C	47.49900436401367	45.62120056152344	56.75358963012695
75	H	48.26367950439453	46.227046966552734	57.23295974731445
76	C	46.861083984375	41.936279296875	56.127647399902344
77	C	45.82493591308594	41.206607818603516	55.42927551269531
78	C	44.53841018676758	40.934532165527344	55.687110900878906
79	H	44.01432418823242	41.48969650268555	56.45804977416992
80	C	43.980865478515625	39.99848937988281	54.84294128417969
81	H	42.982059478759766	39.57468032836914	54.842124938964844
82	C	45.03055191040039	39.54346466064453	54.121089935302734

83	N	46.05782699584961	40.3753547668457	54.32954025268555
84	H	46.96390151977539	40.32557678222656	53.883811950683594
85	C	45.018402099609375	38.34699630737305	53.270118713378906
86	H	44.108150482177734	37.75032424926758	53.30680847167969
87	C	46.05308532714844	37.7003059387207	52.65450668334961
88	C	45.93185806274414	36.592594146728516	51.75688552856445
89	H	44.99430465698242	36.16679763793945	51.41495895385742
90	C	47.24443817138672	36.25642395019531	51.50748062133789
91	H	47.528175354003906	35.51522445678711	50.76765441894531
92	C	48.09388732910156	37.12931442260742	52.22597885131836
93	N	47.3271598815918	38.00287628173828	52.92576217651367
94	C	49.5217399597168	37.49080276489258	52.15253829956055
95	C	50.22187423706055	37.64645767211914	53.30058670043945
96	C	51.44831466674805	37.0518798828125	53.71845626831055
97	H	51.9893913269043	36.29512405395508	53.16008377075195
98	C	51.8453369140625	37.569942474365234	54.924198150634766
99	H	52.76388168334961	37.46794128417969	55.492618560791016
100	C	50.87495040893555	38.55672836303711	55.38106155395508
101	N	50.0228271484375	38.48184585571289	54.3511848449707
102	H	49.26240539550781	39.14590072631836	54.40512466430664
103	C	50.811370849609375	39.31422805786133	56.50899124145508
104	H	51.70225524902344	39.22669219970703	57.12914276123047
105	C	49.74757385253906	40.137760162353516	56.94951629638672
106	C	49.552406311035156	40.88309097290039	58.16684341430664
107	H	50.226905822753906	40.870323181152344	59.01662063598633
108	C	48.41490936279297	41.61381530761719	57.9993896484375
109	H	48.007652282714844	42.337669372558594	58.697532653808594

110	C	47.9850959777832	41.37090301513672	56.69612503051758
111	N	48.76934814453125	40.485355377197266	56.045562744140625
112	C	51.48984146118164	38.401363372802734	48.3558235168457
113	C	52.16487503051758	38.42539978027344	49.575462341308594
114	H	53.19994354248047	38.75377655029297	49.52676010131836
115	C	51.519195556640625	38.17354202270508	50.8245735168457
116	H	52.164424896240234	38.22951126098633	51.697566986083984
117	C	50.180091857910156	37.83189010620117	50.85435104370117
118	C	50.13880157470703	38.13188171386719	48.421417236328125
119	H	49.46516418457031	38.1856689453125	47.57001495361328
120	C	49.500980377197266	37.887447357177734	49.633846282958984
121	H	48.483489990234375	37.504981994628906	49.63750076293945
122	N	52.11379623413086	38.891597747802734	47.1811637878418
123	H	52.85678482055664	38.282005310058594	46.873836517333984
124	C	51.70724105834961	40.00581359863281	46.50692367553711
125	O	50.75913619995117	40.61733627319336	46.92376708984375
126	C	52.56410598754883	40.55735778808594	45.3369026184082
127	C	52.03462600708008	41.6090087890625	44.628143310546875
128	H	51.11983108520508	42.05136489868164	45.01420593261719
129	C	52.67362594604492	42.047245025634766	43.517669677734375
130	C	53.8297004699707	41.482276916503906	43.0191764831543
131	H	54.268062591552734	41.93478775024414	42.13337326049805
132	C	54.31540298461914	40.42294692993164	43.74590301513672
133	H	55.307289123535156	40.023345947265625	43.55083084106445
134	C	53.72775650024414	39.993892669677734	44.92595291137695
135	H	54.172359466552734	39.15231704711914	45.45096969604492
136	C	52.242889404296875	43.328269958496094	42.93733596801758

137	H	51.4394645690918	43.8859977722168	43.41627502441406
138	C	52.69465637207031	43.99808120727539	41.9052734375
139	H	53.344669342041016	43.468841552734375	41.21006393432617
140	C	52.37070083618164	45.31711959838867	41.46882629394531
141	C	52.42784881591797	45.347843170166016	39.95142364501953
142	H	52.42259979248047	44.32730484008789	39.562923431396484
143	H	53.089111328125	46.07210159301758	39.471168518066406
144	H	51.415889739990234	45.63794708251953	39.66118240356445
145	C	52.046630859375	46.320579528808594	42.254005432128906
146	H	51.97414016723633	46.16672134399414	43.32963943481445
147	C	51.6796760559082	47.70216369628906	41.81562805175781
148	H	51.9844970703125	48.155967712402344	40.87378692626953
149	C	51.08934783935547	48.54258346557617	42.64244079589844
150	H	50.830013275146484	48.05810546875	43.5826301574707
151	C	50.659271240234375	49.90117263793945	42.383567810058594
152	C	50.59493637084961	50.319461822509766	40.96383285522461
153	H	51.49571228027344	50.91343307495117	40.795719146728516
154	H	50.605987548828125	49.58332061767578	40.15733337402344
155	H	49.7349853515625	50.97802734375	40.82509231567383
156	C	50.333858489990234	50.6491813659668	43.44314956665039
157	H	50.329124450683594	50.16701126098633	44.41958236694336
158	C	49.90669631958008	52.06046676635742	43.429405212402344
159	H	49.8371696472168	52.557777404785156	42.46308517456055
160	C	49.621612548828125	52.796531677246094	44.55308151245117
161	H	49.780052185058594	52.355892181396484	45.5362663269043
162	C	49.339813232421875	54.23979949951172	44.46391296386719
163	H	49.413665771484375	54.71366882324219	43.486202239990234

164	C	49.17985153198242	55.104652404785156	45.507110595703125
165	C	49.24534606933594	54.70512008666992	46.914005279541016
166	H	48.66777038574219	55.2672233581543	47.65082550048828
167	H	48.73893737792969	53.741661071777344	47.002113342285156
168	H	50.25571060180664	54.511138916015625	47.28007125854492
169	C	49.11373519897461	56.49839401245117	45.403995513916016
170	H	49.393489837646484	57.02169418334961	46.31713104248047
171	C	48.88432693481445	57.19668960571289	44.298397064208984
172	H	48.6845588684082	56.56177520751953	43.43647766113281
173	C	48.92149353027344	58.60444641113281	44.16411209106445
174	H	48.84607696533203	59.18473815917969	45.08253860473633
175	C	48.720157623291016	59.4443473815918	43.12806701660156
176	C	48.56902313232422	58.89438247680664	41.749755859375
177	H	49.23124313354492	58.032100677490234	41.64780044555664
178	H	47.51667404174805	58.610008239746094	41.68525314331055
179	H	48.79799270629883	59.61692810058594	40.96364974975586
180	C	48.542362213134766	60.87748718261719	43.2669563293457
181	H	49.023616790771484	61.23939895629883	44.17433547973633
182	C	47.987281799316406	61.81998825073242	42.45049285888672
183	H	47.45781707763672	61.44569778442383	41.57556915283203
184	C	48.00079345703125	63.195159912109375	42.56373977661133
185	C	49.066585540771484	63.88336944580078	42.92884063720703
186	C	50.46516036987305	63.362648010253906	42.995079040527344
187	H	51.190765380859375	64.09866333007812	42.642601013183594
188	H	50.6693115234375	63.01652145385742	44.01045227050781
189	H	50.60569763183594	62.56560134887695	42.2619743347168
190	C	49.054317474365234	65.4239273071289	43.17162322998047

191	H	49.457584381103516	65.88319396972656	42.266685485839844
192	H	49.69068145751953	65.56209564208984	44.0482177734375
193	C	47.67441940307617	65.90399932861328	43.4544792175293
194	H	47.60163497924805	66.98037719726562	43.6235466003418
195	H	47.31486892700195	65.35801696777344	44.32917785644531
196	C	46.83340835571289	65.48789978027344	42.275455474853516
197	H	45.82044982910156	65.89505004882812	42.30019760131836
198	H	47.348426818847656	65.74227905273438	41.34674072265625
199	C	46.64312744140625	63.93524169921875	42.27799987792969
200	C	45.49209976196289	63.539546966552734	43.17890930175781
201	H	45.7581901550293	63.7509651184082	44.2166748046875
202	H	44.54049301147461	64.05254364013672	43.02481460571289
203	H	45.405338287353516	62.465152740478516	43.00393295288086
204	C	46.019710540771484	63.57357406616211	40.9318733215332
205	H	45.16365051269531	64.17308807373047	40.615318298339844
206	H	46.769439697265625	63.70359802246094	40.14863204956055
207	H	45.757728576660156	62.513797760009766	40.90531539916992
